# Tumor associated microglia/macrophages utilize GPNMB to promote tumor growth and alter immune cell infiltration in glioma

**DOI:** 10.1186/s40478-024-01754-7

**Published:** 2024-04-02

**Authors:** Fatih Yalcin, Hannah Haneke, Ibrahim E. Efe, Leonard D. Kuhrt, Edyta Motta, Bernadette Nickl, Charlotte Flüh, Michael Synowitz, Omar Dzaye, Michael Bader, Helmut Kettenmann

**Affiliations:** 1 Max-Delbrück-Center for Molecular Medicine in the Helmholtz Association, Berlin, Germany; 2 Institute of Pathology, Christian-Albrecht University of Kiel, Kiel, Germany; 3 Department of Neurosurgery, University Medical Center Schleswig-Holstein, Kiel, Germany; 4 Russell H. Morgan Department of Radiology and Radiological Science, Johns Hopkins University, Baltimore, MD USA; 5 DZHK (German Center for Cardiovascular Research), Partner Site Berlin, Berlin, Germany; 6 Charité Universitätsmedizin Berlin, Berlin, Germany; 7 Institute for Biology, University of Lübeck, Lübeck, Germany; 8 Shenzhen Institute of Advanced Technology, Chinese Academy of Sciences, Shenzhen, China

**Keywords:** Microglia, Macrophage, GPNMB, RCAS, Experimental glioma, Mouse, Glioblastoma, CD44

## Abstract

Tumor-associated microglia and blood-derived macrophages (TAMs) play a central role in modulating the immune suppressive microenvironment in glioma. Here, we show that GPNMB is predominantly expressed by TAMs in human glioblastoma multiforme and the murine RCAS-PDGFb high grade glioma model. Loss of GPNMB in the in vivo tumor microenvironment results in significantly smaller tumor volumes and generates a pro-inflammatory innate and adaptive immune cell microenvironment. The impact of host-derived GPNMB on tumor growth was confirmed in two distinct murine glioma cell lines in organotypic brain slices from GPNMB-KO and control mice. Using published data bases of human glioma, the elevated levels in TAMs could be confirmed and the GPNMB expression correlated with a poorer survival.

**Supplementary Information:**

The online version contains supplementary material available at 10.1186/s40478-024-01754-7.

## Introduction

Despite major advances in cancer diagnostics and therapy, the survival rate of high-grade glioma patients has not significantly improved over the last decade. Representing about 80% of malignant primary brain tumors and with an overall annual age-adjusted incidence rate of 3.2 per 100,000, glioblastoma multiforme (GBM) are associated with a high mortality and a current life expectancy of 15 to 17 months after diagnosis, even with therapy. The standard therapy regimen consists of a trimodal treatment with maximal safe surgical resection, temozolomide and concomitant radiotherapy [1, 2]. Advancing GBM therapy regimens through new emerging therapies, such as immunotherapy, remain challenged by a limited understanding of the tumor microenvironment (TME) and especially its highly heterogeneous and immunosuppressive and suppressed innate and adaptive immune cell microenvironment [3, 4].

Tumor-associated microglia and blood-derived macrophages (TAMs) are the most prominent non-neoplastic cell population of GBM, making up to 30–40% of the total tumor mass. Their recruitment to the TME is associated with disease progression and recurrence, poor survival and therapy resistance [5]. TAMs have been established as a significant part and regulator of the immunosuppressive TME due to their anti-inflammatory phenotype, including the self-down regulation of antigen-presentation and secretion of anti-inflammatory cytokines, such as Interleukin-10 (IL-10) and Transforming growth factor β (TGF-ß) [5–7]. Recent studies on experimental glioma models have identified a complex network of signalling molecules by which glioma cells recruit and reprogram microglia and blood-derived macrophages into TAMs to acquire a pro-tumorigenic phenotype. In turn the TAMs release factors by which they promote glioma invasion and expansion [5, 8]. Factors released from glioma cells include extracellular matrix (ECM) components [9], such as versican [10] and tenascin-C (TNC) [11], factors released from TAMs include matrix metalloproteinase-2 (MMP2), matrix metalloproteinase-9 (MMP9) and matrix metalloproteinase-14 (MMP14) 12–14 and the human specific CC motif chemokine ligand 18 (CCL18) [15].

Glycoprotein Nonmetastatic Melanoma Protein B (GPNMB) was identified as a prominent factor upregulated in TAMs of mouse experimental glioma models [16] and human glioma tissue [17, 18]. GPNMB is a type I transmembrane glycoprotein that is overexpressed in several cancers, including glioma [19] and melanoma [20]. Upon expression, GPNMB translocates to the membrane, where proteases, such as A disintegrin and metalloproteinase domain-containing protein 10 (ADAM10), cleave its extracellular domain and shed it into the ECM [21]. The extracellular domain of GPNMB has been described to bind to several receptors, including CD44 on tumor cells [22], stroma cells [23] and astrocytes 24. Additionally, extensive studies on GPNMBs interaction with T cells through Syndecan-4 have demonstrated to impair T cell invasion, activation and proliferation [25–28]. Further receptors for GPNMB are Epidermal Growth Factor Receptor (EGFR), Vascular Endothelial Growth Factor Receptor (VEGFR) as well as integrins and heparins [29].

In tumor-intrinsic knock-down and overexpression models, GPNMB has been described to promote cell growth, angiogenesis and invasion [30, 31]. So far, the relevance of host-derived GPNMB for glioma growth has not been studied in detail, while previous studies have linked to an unfavorable prognosis in glioma patients [32]. We therefore analyzed the role of GPNMB in host-derived glioma expansion. Using the PDGFb-driven replication-competent avian sarcoma-leukosis virus/ tumor virus A (RCAS/Tv-a) murine high-grade glioma model injected into GPNMB-KO and control mice, we demonstrate that host-derived GPNMB is of detrimental importance for glioma growth and immune cell composition.

## Material and methods

### Total RNA isolation and RT-qPCR

Total RNA was isolated using RNA mini kit (Promega, Madison, WI, USA) according to the manufacturer's instructions. Quality and yield were determined by NanoDrop 1000 (PeqLabBiotechnologie, Erlangen, Germany). cDNA was synthesized using 100 ng total RNA with SuperScript II reverse transcriptase kit (Invitrogen, Waltham, MA, USA). RT-qPCR gene amplification was performed in duplicate using SYBR Green PCR mix (Applied Biosystems, Waltham, MA, USA) and 7500 Fast Real-Time PCR System (Applied Biosystems). Primer sequences used were: glycoprotein nonmetastatic melanoma protein B (Gpnmb; murine: sense 5′-AGAAATGGAGCTTTGTCTACGTC-3′, antisense 5′-CTTCGAGATGGGAATGTATGCC-3′; human: sense 5′-TGCGGTGAACCTGATATTCCC-3′, antisense 5′-GTCCTCTGACCATGCTGTCC-3′) and TATA-box binding-protein (Tbp; murine: sense 5′-AAGGGAGAATCATGGACCAG-3′, antisense 5′-CCGTAAGGCATCATTGGACT-3′; human: sense 5′-AGCGCAAGGGTTTCTGGTTT-3′, antisense 5′-CTGAATAGGCTGTGGGGTCA -3′). The results were analyzed by 2^−ΔΔCT^ ways normalized to tbp and were presented as fold change normalized to control group, if not labeled differently.

### Protein extraction and Western Blot

Whole-cell protein extracts were prepared from briefly cultured RCAS-PDGFb (≤ 7 passages) and GL261 glioma cells or primary naïve astrocytes using RIPA lysis buffer (Sigma-Aldrich, St. Louis, MO, USA) containing EDTA-free protease inhibitor cocktail tablets (Roche Diagnostics, Mannheim, Germany). Protein concentration was determined by a BCA protein assay kit (Thermo Fisher Scientific, Waltham, MA, USA), and 20 µg of total protein of each sample was resolved on a 10% SDS-PAGE gel, followed by wet transfer of resolved proteins onto a PVDF membrane (GE Healthcare, Chicago, IL, USA). Membranes were blocked and followed by overnight incubation at 4 °C for murine GPNMB (goat, AF2330; R&D Systems, Minneapolis, MN, USA) and for murine GAPDH (mouse, ab8245; Abcam, Cambridge, UK). Membranes were incubated with a secondary for anti-rabbit HRP antibody at 1:2000 (#7074; Cell Signaling Technology) and for anti-goat with IRDye 680RD (925-68071; Li-Cor, Lincoln, NE, USA), developed with SuperSignal West Pico Chemiluminescence substrate kit (Thermo Fisher Scientific). Signal was detected by Molecular Imager Gel Doc XR system (Bio-Rad, Hercules, CA, USA).

### Immunofluorescent staining and image processing

For murine tumor slices, mouse brains were harvested and perfused with phosphate buffered salt solution (PBS) followed by 4% paraformaldehyde (PFA) solution (Sigma-Aldrich); 40 µm free-floating tumor sections were prepared as previously described [33]. Human glioma specimens were prepared following the procedure previously described [33]. Slices were washed 3 times with PBS for 5 min and blocked with 5% of donkey serum and 0.1% Triton-X (Sigma-Aldrich). Primary antibodies were added overnight 1:100 for murine and human GPNMB (goat; AF2550; R&D Systems), 1:600 for IBA1 (goat; ab5076; Abcam), 1:600 IBA1 (rabbit; ab178847; Abcam), 1:100 for Ki67 (rabbit; ab16667; Abcam), 1:200 for MHCII (rat; 14-5321-81; Invitrogen), 1:100 for CD8A (rat; 14-0081-82; Invitrogen), 1:200 for Gzmb (rabbit; ab4059; Abcam), 1:200 for Foxp3 (rabbit; mAb #12653; Cell Signaling), 1:100 for CD3 (rat; 14-0032-81; Invitrogen), 1:200 for PD-1 (rabbit; ab214421; Abcam), 1:200 for CD44 (rabbit; 14-0441-81; Invitrogen) at 4 °C. As secondary antibody, we used 1:200 anti-rabbit IgG (711-545-152; Dianova, Hamburg, Germany), anti-goat IgG (705-605-147; Dianova), and anti-rat IgG (712-545-153; Dianova), 1:200 anti-rat IgG (Cy5-labelled; 712-175-150; Jackson Immuno Research, Cambridgeshire, United Kingdom) and anti-rabbit IgG (Cy3-labelled; 711-165-152 Dianova). Nuclei were counterstained with 4,6-diamidino-2-phenylindole (DAPI; Sigma-Aldrich). Images were taken using a confocal microscope (LSM710 & LSM700; Carl Zeiss, Oberkochen, Germany) with 10×, 20×, 40× or 63× oil objectives. Cell counting and area of staining was performed using Imaris software (Bitplane, Zürich, Switzerland) and Fiji [34]. For human tumor and control slices, tissue was cut at 5 µm sections and floated onto charged slides for preparation of immunohistochemistry.

### Animals

Mice were housed in the animal facilities of the Max Delbrueck Center and handled according to governmental guidelines (LaGeSo G125/17). GPNMB−/− (GPNMB-KO; KO) mice were generated as described [35]. The first base after the start codon ATG of the gene GPNMB was deleted with Crispr-Cas9 technology in the C57BL/6N background strain (SI Methods). KO mice were held homozygously knockout for GPNMB alleles and were compared to the C57BL/6N (wildtype; WT) strain (Charles River, Sulzfeld, Germany) in animal experiments.

### Generation of intracranial mouse gliomas

Ntv-a/Ink4a-Arf−/− mice develop pro-neural high-grade gliomas 6–8 weeks following intracranial injection of RCAS-PDGFb-producing DF-1 chicken fibroblast cells at 4.5–10 weeks of age [36]. RCAS-PDGFb tumors were dissociated as described below and intracranially re-transplanted into KO and WT mice. Injections were performed using a stereotactic frame (Stoelting, Wood Dale, IL, USA). Mice used for these experiments were 5–10-week-old (Ntv-a/Ink4a-Arf−/− mice for DF-1 RCAS-PDGFb injection), or 8–14-week-old (C57BL/6N WT or KO for RCAS-PDGFb tumor cell re-implantation). Mice were anesthetized with intraperitoneal injections of 0.1 mg/g ketamine (Pharmazeutischen Handelsgesellschaft, Garbsen, Germany) and 0.02 mg/g xylazine (Bayer, Leverkusen, Germany). Animals were also provided 0.25% Marcaine in the volume of about 0.1 ml/25 g administered right before the surgery, which provided pain relief from the sutures for 6–8 h. One microliter cell suspension (4 × 10^4^ transfected DF-1 cells, or 5 × 10^4^ RCAS-PDGFb tumor cells) was delivered using a 30-gauge needle attached to a Hamilton syringe (Hamilton; Reno, NV, USA). Coordinates for injections of DF-1 cells into Ntv-a/Ink4a-Arf−/− mice and RCAS-PDGFb tumor cells into WT or KO mice, respectively were bregma 1.5 mm anterior, Lat − 0.5 mm, and a depth 2.0 mm. Tumors for the GL261 mouse model were implanted as previously described [16].

### Analysis of disease progression

RCAS-PDGFb tumor-bearing wild type and KO mice were monitored for signs of disease progression using a score from 1 (no symptoms) to 5 (severe disease). Score 1: No neurological deficit; when lifting by the tail, front paws remain on the ground and animal resists strong pull. Score 2: Shaggy coat with persistent grooming; when lifting by the tail, front paws remain on the ground and animal resists moderate pulling. Score 3: Weight loss 5%; unsteady walking pattern; when lifting by the tail, front paws remain on the ground and animal resists slight pull. Score 4: Increasing weight loss up to 15%; delayed reaction to touch and sounds; resistance of the front paws when lifting not present; low curvature of the back. Score 5: Eyes mostly closed; blindness; weight loss 20% of initial weight; no reaction to touch or sound; hunchback. A mouse was euthanized whenever it reached a score between 3 and 4. Monitoring of the mice occurred daily; weighing and behavioral assessment reflecting the mice’s health condition started on day 21 post surgery.

### Tumor volume quantification

WT and KO mice were inoculated with RCAS-PDGFb cells and sacrificed together whenever the first mouse reached a disease score between 3 and 4. Brains were fixed after perfusion and cut into 40 μm thick slices. Slices were stained with DAPI and antibodies against IBA1 and GPNMB. The tumor volume was reconstructed through the Cavalieri method by measuring the RFP^+^ area in every 12th tumor slice.

### Bioinformatic analysis (TCGA, CGGA)

GlioVis (http://gliovis.bioinfo.cnio.es) was used to access tumor gene expression data of GBM patients for GPNMB, macrophage and glioma/astrocyte marker genes (Study: Glioblastoma, mRNA Expression z-scores HG-UG133A. The dataset from Klemm at el. [18] was made available by the authors through their open access platform: https://joycelab.shinyapps.io/braintime/. For correlationship evaluation between TAMs infiltration level and gene expression, TCGA GBM RNA-seq datasets were processed via TIMER platform (http://timer.comp-genomics.org/) [37].

### Human material

All patients were operated at the Department of Neurosurgery, University Medical Center Schleswig–Holstein, Campus Kiel. The study was approved by the Ethics Committee of the University of Kiel (approval #D477/18) and was in accordance with the Helsinki Declaration of 1964 and its later amendments. Informed consent was obtained from all individual patients. For non-tumor samples, tissue resected from trauma patients undergoing brain surgery were used. Freshly resected tumor and non-tumor tissue was stored in Dulbecco’s modified Eagle’s medium (DMEM; Invitrogen, Carlsbad, CA, USA) at 4 °C for < 24 h until further experimental workup. Samples were either fixed in 4% PFA for 24 h and mounted in tissue tec at − 20 °C or prepared for Cell sorting via MACS isolation (described below).

### Cell isolation

In tumor-bearing mice brains, only the visible tumor area around the injection site was used. Human tissue was dissociated with the Brain Tumor Tissue Dissociation Kit (Miltenyi Biotec) according to the manufacturer’s instructions. Subsequently, the cell pellet was resuspended in sorting buffer for subsequent magnetic-activated cell sorting (MACS).

Murine tissue was mechanically dissociated as described previously [38]. To remove myelin we followed a protocol published by Guneykaya et al. [39] In brief, the brain cell suspension was mixed with a total of 25 ml of a 22% Percoll (Th.Geyer, Renningen, Germany) solution and a layer of 5 ml cold PBS (Gibco-Invitrogen) was added on top. Centrifugation at 950 g with slow acceleration and without breaks created a gradient that separated the cell pellet on the bottom of the tube from the myelin which was carefully aspirated. For the isolation of TAMs from RCAS-PDGFb tumors-bearing WT mice a 30%/70% Percoll gradient was used. After 25 min of centrifugation at 800 g TAMs and naïve microglia were enriched at the 30%/70% interphase. Cells were collected, washed once with PBS, and subsequently centrifuged again at 300 g for 10 min. Subsequently, the cell pellet was resuspended in sorting buffer for subsequent flow-cytometry.

Spleens were processed through a 70 μm cell strainer with a syringe plunger and the mesh rinsed with 10 ml of PBS per spleen. The cells were centrifuged and the pellet subjected to erythrocyte lysis by adding 5 ml of 1 × RBC lysis buffer (Biolegend, San Diego, CA, USA). The lysis was carried out by shaking the tube mildly for 5 min at RT and subsequently stopped with 20 ml of PBS. The pellet was washed once with PBS and resuspended in PBS, containing 0.5% FCS and 2 mM EDTA (FACS buffer) for subsequent fluorescence activated cell sorting (FACS) isolation.

### MACS sorting

The CD11b^+^ samples for the microarray were generated using MACS. Following Percoll gradient centrifugation, tumor and control cell pellets were resuspended in PBS, containing 0.5% fetal calf serum (FCS) and 2 mM EDTA and labeled with anti-CD11b microbeads (Miltenyi Biotec). The MACS isolation was carried out according to the manufacturer’s instructions and cells were subsequently used for RNA isolation.

### FACS analysis

FACS was employed using CD11b, CD45, Ly6c, Ly6G and GPNMB to determine the GPNMB^+^ population of non-immune cells (CD45^−^CD11b^−^), microglia or TAMs (CD45^+^CD11b^+^Ly6G^−^Ly6c^−^), monocytes with low Ly6c expression (CD45^+^CD11b^+^Ly6G^−^Ly6c^low^), monocytes with high Ly6c expression (CD45^+^CD11b^+^Ly6G^−^Ly6c^high^), neutrophils (CD45^+^CD11b^+^Ly6G^+^Ly6c^+^) and lymphocytes (CD45^+^CD11b^−^). Following Percoll gradient centrifugation, cell pellets were resuspended in FACS buffer (containing 2% FCS) and stained with 2 μl of dye-coupled antibodies per 1 × 10^7^ cells. The staining was performed with CD45-eFluor450 (48-0451-82), CD11b-Pe-Cyanine7 (25-0112-82), Ly6c-PE (12-5932-80), Ly6G-FITC (11-5931-82), and GPNMB-eFluor660 (50-5708-82; all eBioscience, San Diego, CA, USA) for 30 min at 4 °C. Thereafter, the cells were washed and resuspended in 500 μl FACS buffer per 5 × 10^6^ cells for sorting at a Fortessa (BD Bioscience). Compensation was calculated with single-stained beads (552844; BD Bioscience, Franklin Lakes, NJ, USA) and unstained cells.

### Cell culture

Cells of the murine glioma cell line GL261 (National Cancer Institute, MD, USA) were grown in DMEM with 10% FCS, 200 mM glutamine, 100 U/ml penicillin, and 100 mg/ml streptomycin (all from Invitrogen). DF-1 cells were purchased from ATCC (Manassas, VA, USA). Cells were grown at 39 °C according to ATCC instructions. Transfections with RCAS-PDGFb were performed using Fugene 6 transfection kit (no. 11814443001; Roche, Mannheim, Germany) according to manufacturer’s protocol. Primary microglia were prepared from neonatal and adult C57BL/6 as previously described [12]. Briefly, cortical tissue of neonatal mice was freed of blood vessel and meninges in Hank’s balanced salt solution (HBSS), and digested in 1% trypsin and 0.05% deoxyribonuclease for 5 min at room temperature. Cells were then plated in 75-cm^2^ flasks. After 7 days, cells were treated with L929 conditioned medium. Microglia were then shaken off and replated. All cells were maintained in a 37 °C incubator with a 5% CO_2_ humidified atmosphere.

### Tumorsphere culture

RCAS-PDGFb tumors were excised from tumor brains using a scalpel, minced, and incubated with Accutase (eBioscience, San Diego, CA, USA) for 15 min at 37 °C. Tissue pieces were mechanically dissociated using a 1 ml pipette and washed in DMEM. Cells were passed through a 70 μm cell strainer and seeded into a T25 cell culture flask. Cells were grown in GIC medium containing DMEM-F12 GlutaMAX (GIBCO-Invitrogen, Carlsbad, CA, USA), 1% penicillin G/streptomycin sulfate (Sigma-Aldrich), 1:50 B-27 without vitamin A (GIBCO-Invitrogen), 0.2 mM HEPES (Sigma-Aldrich), 20 ng/ml insulin (Sigma-Aldrich), supplemented with 20 ng/ml fibroblast growth factor 2 (FGF2; Cell Systems, Kirkland, WA, USA) and 20 ng/ml epidermal growth factor (EGF; Cell Systems).

### Organotypic brain slice (OBS) model and tumor inoculation

OBSs were prepared as described previously [15]. Briefly, 14-day-old WT or KO mice were decapitated, and brains were cut in coronal plane into 250 µm sections with a vibratome (Leica Microsystems, VT1000S, Wetzlar, Germany). Brain slices were collected with a sterile plastic pipette (7 mm diameter) and transferred onto cell culture inserts with 0.4 µm pores (Becton Dickinson, Franklin Lakes, NJ, USA), which were fitted into wells of a 6-well plate; 1 ml of culture medium containing DMEM supplemented with 10% heat-inactivated FCS, 0.2 mM glutamine, 100 U/ml penicillin, and 100 mg/ml streptomycin was added into each well; after overnight incubation, medium was changed with cultivation medium containing 25% heat-inactivated FCS, 50 mM sodium bicarbonate, 2% glutamine, 25% Hanks balanced salt solution, 1 mg/ml insulin (Invitrogen), 2.46 mg/ml glucose (Braun, Melsungen, Germany), 0.8 mg/ml vitamin C (Sigma-Aldrich), 100 U/ml penicillin, 100 mg/ml streptomycin, and 5 mM Tris in DMEM (all from Invitrogen). 5000 mCherry-GL261 or RFP-labelled RCAS-PDGFb cells were slowly injected into the caudate putamen region of the slice in 150 µm depth of both hemispheres using a Hamilton syringe. Careful control of the injection procedure ensured that no cells spilled onto the surface of the slice, which could migrate over the surface rather than invade through the tissue. After 4 days, mCherry-GL261 or, after 6 days, RFP-labelled RCAS-PDGFb slices were washed and fixed with 4% PFA. Tumor volumes were measured by confocal microscopy (LSM710; Carl Zeiss) with z-stack scanning and were reconstructed by Imaris into 3D model for exact volume evaluation.

### Softwares and statistics

Graphs were created using GraphPad Prism 7 & 10 and were analyzed using an unpaired parametric 2-tailed t-test, assuming equal standard deviations. One-way ANOVA was used in experiments having more than one group to compare with controls. Linear regression was used to compare curves of disease progression. Test details are included in appropriate figure legends. Pearson correlation was calculated using GraphPad Prism 7. Imaris Version 9.3.1 was used.

## Results

### Depletion of host-derived GPNMB impairs glioma formation and proliferation in vivo

**Fig. 1:**
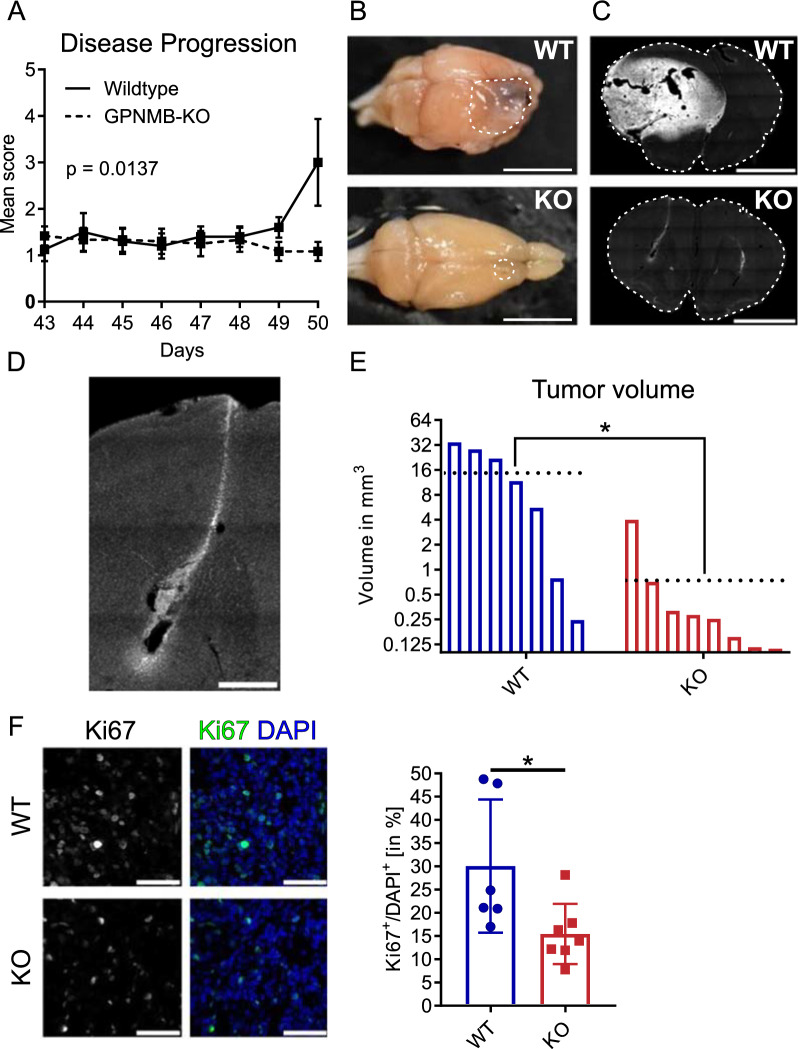
Depletion of host-derived GPNMB impairs glioma formation and proliferation in vivo. **A** Disease progression score of tumor-bearing WT and KO mice injected with PDGFb-driven RCAS-RFP glioma cells. Animals were sacrificed upon the first animal reaching disease score 3 (day 50). **B** Representative macroscopic image of a tumor-bearing brain from WT (top) and KO (bottom) mice. The white dotted line marks the tumor in WT and in KO. Scale bars represent 5 mm. **C** RFP^+^ fluorescence (grey scaled for visibility) labeling of glioma cells of a representative brain slice from a tumor-bearing WT (top) and KO (bottom) mice. The white dotted line marks the whole brain tissue. Scale bars represent 2 mm. **D** RFP^+^ fluorescence (grey scaled for visibility) of tumor cells at the injection site of a representative KO brain slice. Scale bar represents 500 µm. **E** Tumor volume of tumor-bearing WT (n = 7) and KO (n = 8) mice. Black dotted line represents the mean value of each group. Statistical analysis was performed using unpaired t-test. **F** Representative staining of Ki-67 (left) and merged with DAPI (right; Ki-67 = green, DAPI = blue) of brain slices from tumor-bearing WT (n = 6) and KO (n = 7). Scale bar represents 50 µm. Statistical analysis was performed using an unpaired t-test. Error bars represent SD. *p < 0.05

We used a genetically engineered mouse model of adult PDGFb-driven gliomas based on RCAS/Tv-a to implant primary RFP-labeled glioma cells into KO (n = 8) and respective WT control mice (n = 7), to determine the impact of host-derived GPNMB for glioma formation and growth. Starting on day 50 after glioma injection, control mice showed symptoms such as fatigue, lethargy, ungroomed fur and curved posture, while the KO mice were symptom-free (*p* = 0.0137; Fig. 1A). All animals were euthanized on that day and the brains were isolated. Concordantly to their absence of symptoms, KO mice had no macroscopically visible tumors or abnormalities of the brain parenchyma, whereas WT mice showed distinct signs of hemorrhage, stroma retraction and necrosis (Fig. 1B). The tumor volume was determined with the Cavalieri method (tumor area evaluated from every 12th slice) based on the RFP fluorescently labeled glioma cells (Fig. 1C) and confirmed by higher nuclear density in DAPI (Additional file 1; Fig. 1A). In KO mice, RFP^+^ glioma cells were predominantly only detected in and around the injection canal (Fig. 1D, Additional file 1: Fig. 1B), while tumor in the WT mice were disseminated throughout the hemisphere, even in the relative smaller WT tumors (Additional file 1; Fig. 1C).

The mean size of tumors in WT was 14.69 mm^3^, while the mean size of the tumors in the KO was 0.74 mm^3^ and thus 19-times smaller (*p* = 0.0126; Fig. 1E). Note that the values for the tumor sizes in Fig. 1E are on a logarithmic scale.

To quantify the proliferative activity, we stained slices containing tumor tissue of both groups for the proliferation marker Ki67 and DAPI. KO displayed around 50% less Ki67^+^ cells normalized to DAPI + cells (equivalent of the entire cell population), compared to tumor-bearing WT mice (WT: n = 6; mean = 30.07% ± 14.33 SD; KO: n = 7; mean = 15.45% ± 6.48 SD; p = 0.033; Fig. 1F).

### GPNMB is upregulated in the murine TME and is predominantly expressed by TAMs

**Fig. 2:**
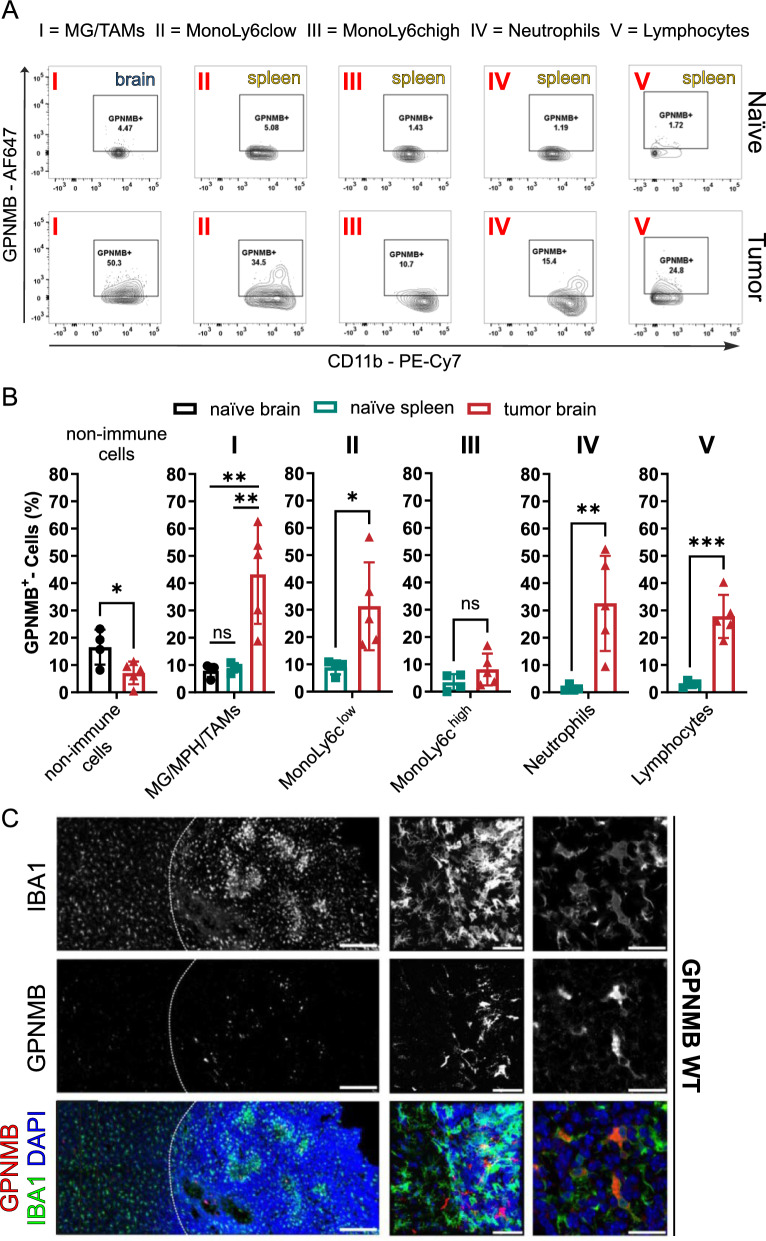
GPNMB is upregulated in the murine TME and is predominantly expressed by TAMs. **A** Representative FACS analysis of GPNMB^+^ populations in MG/TAMs (I), MonoLy6c^low^ (II), MonoLy6c^high^ (III), neutrophils (IV) and lymphocytes (V) in naïve brain and spleen tissue (top row) and tumor-bearing brain tissue (bottom row). **B** Quantification of GPNMB expression in non-immune cells (CD45^−^CD11b^−^). Comparison of naïve brain (n = 4) against tumor-bearing (n = 5) brain tissue. GPNMB expression in microglia (MG)/macrophages (MPH)/TAMs (I; CD45^+^CD11b^+^Ly6G^−^Ly6c^−^). Comparison between naïve brain (n = 4), naïve spleen (n = 4) and tumor brain (n = 5). GPNMB expression in monocytes with low Ly6c (II; CD45^+^CD11b^+^Ly6G^−^Ly6c^low^), monocytes with high Ly6c (III; CD45^+^CD11b^+^Ly6G^−^Ly6c^high^), neutrophils (IV; CD45^+^CD11b^+^Ly6G^+^Ly6c^+^) and lymphocytes (V; CD45^+^CD11b^−^). Comparison of naïve spleen (n = 4) against tumor-bearing (n = 5) brain tissue. Error bars represent SD. Statistical analysis in 3 groups was performed using Ordinary one-way ANOVA with Tukey’s multiple comparisons test and in 2 groups using unpaired t-test. *p < 0.05, **p  < 0.01, ***p < 0.001, ns, not significant. **C** Representative brain slices from tumor-bearing WT mice stained for IBA1 (top row), GPNMB (middle row) and merge with DAPI (bottom row; IBA1 = green, GPNMB = red, DAPI = blue). Left column: Lower magnifications of the invasive edge (white dotted line = invasive edge; left: brain tissue; right: tumor tissue). Scale bar represents 200 µm. Middle column: Higher magnifications of the invasive edge. Scale bar represents 50 µm. Right column: Representative GPNMB^+^/IBA1^+^ cells in the tumor core. Scale bar represents 20 µm

To determine whether GPNMB was upregulated in the murine TME, we used flow-cytometry to detect extracellular membrane-bound GPNMB of isolated cells from the tumor region of a separate WT mice cohort (n = 6) injected with unlabelled RCAS-PDGFb glioma cells. In 5 out 6 mice, a macroscopic distinguishable tumor (n = 5) was detected. We compared GPNMB expression of TAMs with microglia from age-matched naïve animals and separated them from non-immune cells (CD45^−^CD11b^−^) which are either predominantly glioma cells in the tumor tissue or neurons and macroglia in the normal brain tissue. Monocytes with low Ly6c expression (CD45^+^CD11b^+^Ly6G^−^Ly6c^low^), monocytes with high Ly6c expression (CD45^+^CD11b^+^Ly6G^−^Ly6c^high^), neutrophils (CD45^+^CD11b^+^Ly6G^+^Ly6c^+^) and lymphocytes (CD45^+^CD11b^−^) in the tumor tissue were compared with the corresponding population isolated from the spleen of naïve animals (n = 4). The detailed protocol of the FACS isolation protocol is shown in Additional file 2; Fig. 2A. Mean percentage of the CD45^+^CD11b^+^ population in the naïve brain was 94.9% microglia/TAMs, 0.6% MonoLy6c^low^, 0.4% MonoLy6c^high^ and 3,8% neutrophils, while in the brain with tumor cells we found 75.7% microglia/TAMs, 10.5% MonoLy6c^low^, 9% MonoLy6c^high^ and 3.5% neutrophils. Taken together, in the innate immune cell compartment, a higher percentage of monocytes was detected in the TME (Additional file 2; Fig. 2B). In Fig. 2A we show an example of the isolation of the microglia and TAM population from brain and the comparison of the population of monocytes with low and high Ly6c expression, neutrophils and lymphocytes from glioma tissue with the cells from spleen.

Based on the FACS isolation, we quantified the populations of GPNMB expressing cells (Fig. 2B). In comparison to our naïve control brain tissue where the CD45^−^CD11b^−^ represent macroglia and neurons, we detected a significantly lower percentage of GPNMB^+^ cells in non-immune cells of tumor-bearing mice, which predominantly represents the glioma cells (naïve brain = 16.59% ± 6.4 SD vs. tumor brain = 7.12% ± 4.17 SD; *p* = 0.0309) indicating that the glioma cells express less GPNMB than macroglia and neurons. Comparing the percentage of GPNMB expressing TAMs to CD45^+^CD11b^+^Ly6G^−^Ly6c^−^ cells in naïve brain representing microglia (naïve brain = 7.98% ± 2.37 SD vs. tumor brain = 43.2% ± 18.09 SD; *p* = 0.0028) and naïve spleen representing macrophages (naïve spleen = 8.98% ± 1.7 SD vs. tumor brain = 43.2% ± 18.09 SD; *p* = 0.0034), we found significantly higher GPNMB levels in TAMs, indicating that the TME induces an upregulation of GPNMB expression in TAMs. No difference was found between microglia in the naïve brain and naïve spleen macrophages (p = 0.9917). Blood-derived monocytes with high Ly6c expression showed no significant difference between the naïve spleen and TME (naïve spleen = 3.44% ± 3.01 SD vs. tumor brain = 8.14% ± 5.85 SD; p = 0.1918). In contrast, there were fewer GPNMB^+^ cells among monocytes with low Ly6c expression in the naïve spleen versus tumor tissue (naïve spleen = 8.87% ± 2.55 SD vs. tumor brain = 31.28% ± 16.09 SD; p = 0.0297). Furthermore, a higher percentage of neutrophils in the TME significantly expressed GPMNB compared to neutrophils isolated from naïve spleens (naïve spleen = 1.52% ± 1.08 SD vs. tumor brain = 32.6% ± 17.41 SD; p = 0.0098). Interestingly, lymphocytes display also a consistent increase of GPNMB in the TME (naïve spleen = 3.02% ± 1.10 SD vs. tumor brain = 27.8% ± 7.89 SD; p = 0.0005).

To further characterize and validate the expression of GPNMB in the TME, we stained brain slices of GPNMB WT mice implanted with RCAS-PDGFb tumors for GPNMB, IBA1 and DAPI. Expression of GPNMB was detected predominantly within the tumor margin (in the area with RFP^+^ cells), but not outside the tumor margin. In the tumor core, GPNMB was predominantly expressed by IBA1^+^ cells and only few IBA1^−^ cells. At the invasive edge of the tumor, we observed higher levels of GPNMB, which were expressed by ameboid IBA1^+^ cells. IBA1^+^ cells at a distance from the tumor were ramified and had no detectable GPNMB expression (Fig. 2C).

Taken together, these data suggest a high level of GPNMB expression in immune cells including TAMs within the TME of the RCAS-PDGFb model.

### Non-tumor, adult GPNMB-KO mice display a higher density and soma size of microglia

**Fig. 3:**
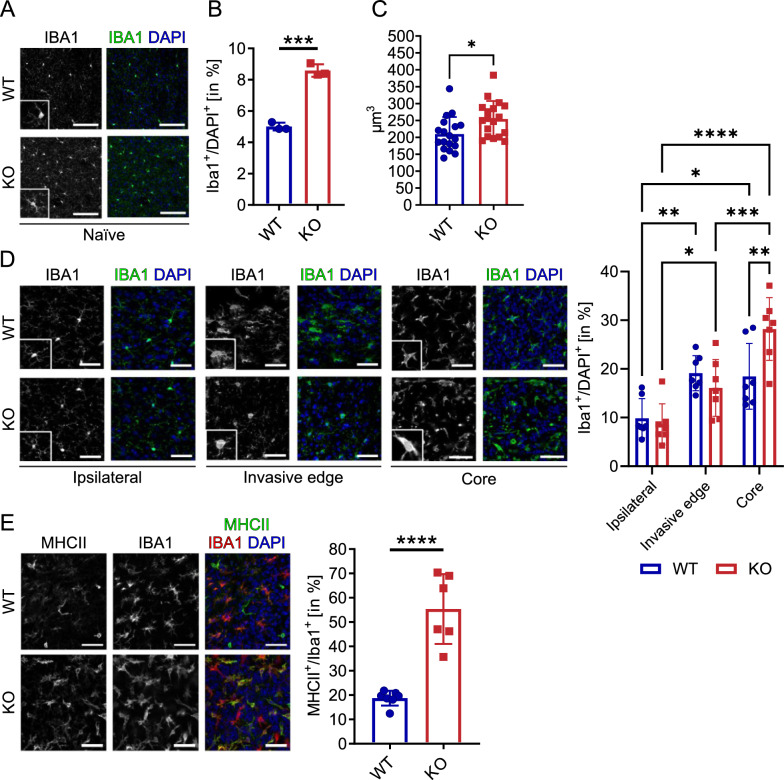
GPNMB-KO mice display a higher density and soma size of microglia and accumulate pro-inflammatory TAMs. **A** Representative staining of IBA1 (left) and merge with DAPI (right; IBA1 = green, DAPI = blue) of brain slices from naïve WT (n = 3) and KO (n = 3). Scale bar represents 100 µm. The inserts show an expanded magnification of an individual microglia. **B** IBA1^+^ cell density normalized to DAPI in % and **C** soma volume (in µm^3^) as obtained from slices as shown in A. Statistical analysis was respectively performed using unpaired t-test. **D** Representative stainings of IBA1 (left) and merge with DAPI (right; IBA1 = green, DAPI = blue) of brain slices from tumor-bearing WT (n = 7) and KO (n = 7) in three different brain regions. Ipsilateral: Outside of the tumor in the ipsilateral hemisphere. Invasive edge (IE): Border region from tumor to non-tumor brain tissue, defined by the RFP signal and DAPI density. Core: Core tumor tissue area. Scale bar represents 40 µm. The inserts show an expanded magnification of an individual microglia. The bar graph on the right summarizes the percentage of IBA1^+^ cells normalized to the DAPI^+^ cells for these three tissue regions. Statistical analysis was performed using 2-way ANOVA with Sidak´s multiple comparisons test. **E** Representative staining of MHCII (left), IBA1 (middle) and merge with DAPI (right; MHCII = green, IBA1 = red, DAPI = blue) of brain slices from tumor-bearing WT (n = 7) and KO (n = 6). Scale bar represents 50 µm. Statistical analysis was performed using an unpaired t-test. Error bars represent SD. *p < 0.05, **p < 0.01, ***p < 0.001, ****p < 0.0001

We studied the microglia density and soma volume in cortical brain slices from KO (n = 3) and WT mice (n = 3). To determine microglial density, we stained the slices with anti-IBA1-antibodies and DAPI. We found that the density of microglial cells in relation to all DAPI^+^ cells was higher in cortical slices from KO compared to WT animals (WT = 5.01% ± 0.25% SD vs. KO = 8.59% ± 0.4% SD; p = 0.0002; Fig. 3A, B). This effect was consistent counting only microglial cells in each selected frame (WT = 17 ± 0.58 SD vs. KO = 27 ± 3.04 SD; *p* = 0.0057; Additional file 3; Fig. 3A). The soma volume of microglia in slices from KO mice was 21% larger as compared to WT animals (WT = 209.8 μm^3^ ± 50.73 μm^3^ vs. KO = 254.6 μm^3^ ± 53.07 μm^3^ SD; p = 0.0142; Fig. 3C).

### GPNMB-KO mice show a higher density of TAMs in the tumor core and a higher level of MHCII expression

To assess the density of IBA1^+^ cells in the TME, we compared the density of IBA1^+^ cells in the non-tumor areas of the ipsilateral hemisphere, at the invasive edge and the tumor core and in addition at the contralateral site. The IBA1^+^ cell density in the contralateral hemisphere of the WT´s did not significantly differ compared to naïve WT animals (5.62% ± 1.42% SD; Additional file 3; Fig. 3C).

At the ipsilateral site, compared to naïve mice, we found a higher density of IBA1^+^ cells both in the KO (KO = 8.62% ± 4.21%, n = 7), but also in the WT animals (WT ipsilateral = 9.85% ± 4.03% SD; n = 7). The difference was not significant (p = 0.9627; Fig. 3D). At the invasive edge, the density was higher compared to the ipsilateral hemisphere, but there was also no difference between WT and KO animals (WT = 19.12% ± 3.59% SD vs. KO = 16.08% ± 5.83% SD; *p* = 0.6407). However, a significant higher density of TAMs was found in the tumor core (*p* = 0.0045) of KO mice (28.19% ± 6.45% SD) compared to WT (18.47% ± 6.76% SD). Interestingly, while there was no difference in the density of IBA1^+^ cells between the invasive edge and core of WT tumors (WT invasive edge = 19.12% ± 3.59% SD vs. WT core = 18.47% ± 6.76% SD; p = 0.99), the IBA1^+^ cell density was significantly higher by 75% comparing the invasive edge with the core in KO mice (KO invasive edge = 16.08% ± 5.83% SD vs. KO core = 28.19% ± 6.45% SD; *p* = 0.0004; Fig. 3D).

To elucidate the antigen presentation capabilities of the TAMs in the tumor tissue, we labelled the major histocompatibility complex II (MHCII), IBA1 and DAPI, to determine the MHCII^+^/IBA1^+^ population of TAMs in WT (n = 7) and KO (n = 6) mice. We counted 2.95 times more MHCII^+^/IBA1^+^ cells (normalized to the number of DAPI cells) in the KO tissue compared to WT (WT = 18.75% ± 3.03% SD vs. KO = 55.38% ± 14.32% SD; *p* ≤ 0.0001; Fig. 3E).

### Loss of host-derived GPNMB promotes a pro-inflammatory tumor immune microenvironment

**Fig. 4:**
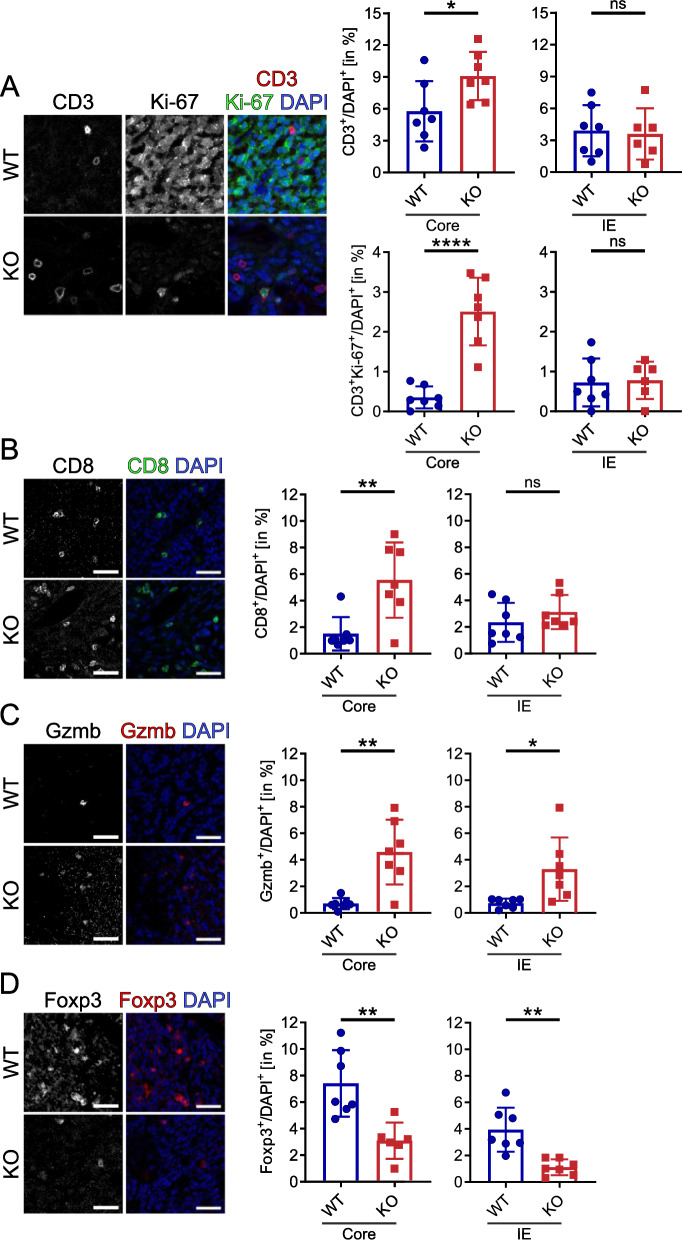
Loss of host-derived GPNMB promotes a pro-inflammatory tumor immune microenvironment in a murine glioma model. **A** Representative core region staining of CD3 (left), Ki-67 and merge with DAPI (right; CD3 = red, Ki-67 = green, DAPI = blue) of brain slices from tumor-bearing WT and KO. Scale bar represents 20 µm. The graphs (Top left, core, CD3^+^DAPI^+^: WT n = 7, KO n = 7; Top right, invasive edge (IE), CD3^+^DAPI^+^: WT n = 7, KO n = 7; Bottom left, core, CD3^+^Ki-67^+^DAPI^+^: WT n = 7, KO n = 7; Bottom right, IE, CD3^+^Ki-67^+^DAPI^+^: WT n = 7, KO n = 6) show the summary data from WT and KO mice. **B** Representative core region staining of CD8 (left) and merge with DAPI (right; CD8 = green, DAPI = blue) of brain slices from tumor-bearing WT and KO. Scale bar represents 40 µm. The graphs (left, core: WT n = 7, KO n = 7; right, IE: WT n = 7, KO n = 7) show the summary data from WT and KO mice. **C** Representative core region staining of Gzmb (left) and merge with DAPI (right; Gzmb = red, DAPI = blue) of brain slices from tumor-bearing WT and KO. Scale bar represents 40 µm. The graphs (left, core: WT n = 7, KO n = 7; right, IE: WT n = 7, KO n = 7) show the summary data from WT and KO mice. **D** Representative core region staining of Foxp3 (left) and merge with DAPI (right; Foxp3 = red, DAPI = blue) of brain slices from tumor-bearing WT and KO. Scale bar represents 40 µm. The graphs (left, core: WT n = 7, KO n = 7; right, IE: WT n = 7, KO n = 7) show the summary data from WT and KO mice. Statistical analysis was performed using an unpaired t-test. Error bars represent SD. *p < 0.05, **p < 0.01, ***p < 0.001, ****p < 0.0001

To further assess the impact of GPNMB on immune cell composition in the glioma tissue, we stained tumor slices for CD3 labelling T cells in general and combined with Ki-67 labelling the respective proliferating subpopulation, for CD8 labelling CD8^+^ T cells, for Granzyme B (Gzmb) labelling Granzyme B^+^ cytotoxic cells and for Foxp3 labelling regulatory T cells. DAPI co-staining was used to relate the populations to the total number of cells. Quantification was performed in the tumor core and at the invasive edge. In the core region of the KO tumors, we detected higher infiltrates of CD3^+^ T cells (WT = 5.76% ± 2.84% SD vs. KO = 9.08% ± 2.27% SD; *p* = 0.0325), proliferating CD3^+^Ki-67^+^ T cells (WT = 0.35% ± 0.28% SD vs. KO = 2.51% ± 0.85% SD; *p* ≤ 0.0001), CD8^+^ T cells (WT = 1.51% ± 1.26% SD vs. KO = 5.55% ± 2.835% SD; *p* = 0.0048) and Granzyme B^+^ cytotoxic cells (WT = 0.7% ± 0.42% SD vs. KO = 4.59% ± 4.59% SD; *p* = 0.0013) compared to the tumor tissue of WT. Additionally, we found a lower density of Foxp3^+^ regulatory T cells in glioma tissue of KO versus WT (KO = 3.09% ± 1.37% SD; WT = 7.41% ± 2.5 SD: p = 0.0031; Fig. 4A-C).

In the invasive edge (IE) of the KO tumors, we detected similar to the core higher infiltrates of Granzyme B^+^ cytotoxic cells (WT = 0.74% ± 0.35% SD vs. KO = 3.3% ± 2.39% SD; p = 0.0161) and a lower density of Foxp3^+^ regulatory T cells (WT = 3.95% ± 1.66% SD vs. KO = 1.11% ± 0.60% SD; p = 0.0011) compared to the invasive edge of WT. No significant difference was found here for CD3^+^ T cells (WT = 0.72% ± 0.6% SD vs. KO = 0.78% ± 0.47% SD; p = 0.86), proliferating CD3^+^Ki-67^+^ T cells (WT = 3.9% ± 2.41% SD vs. KO = 3.59% ± 2.42% SD; p = 0.82) and CD8^+^ T cells (WT = 2.35% ± 1.47% SD vs. KO = 3.13% ± 1.29% SD; p = 0.31).

### Loss of host-derived GPNMB impairs tumor growth in OBS cultures injected with glioma cells with low and high intrinsic expression of GPNMB

**Fig. 5:**
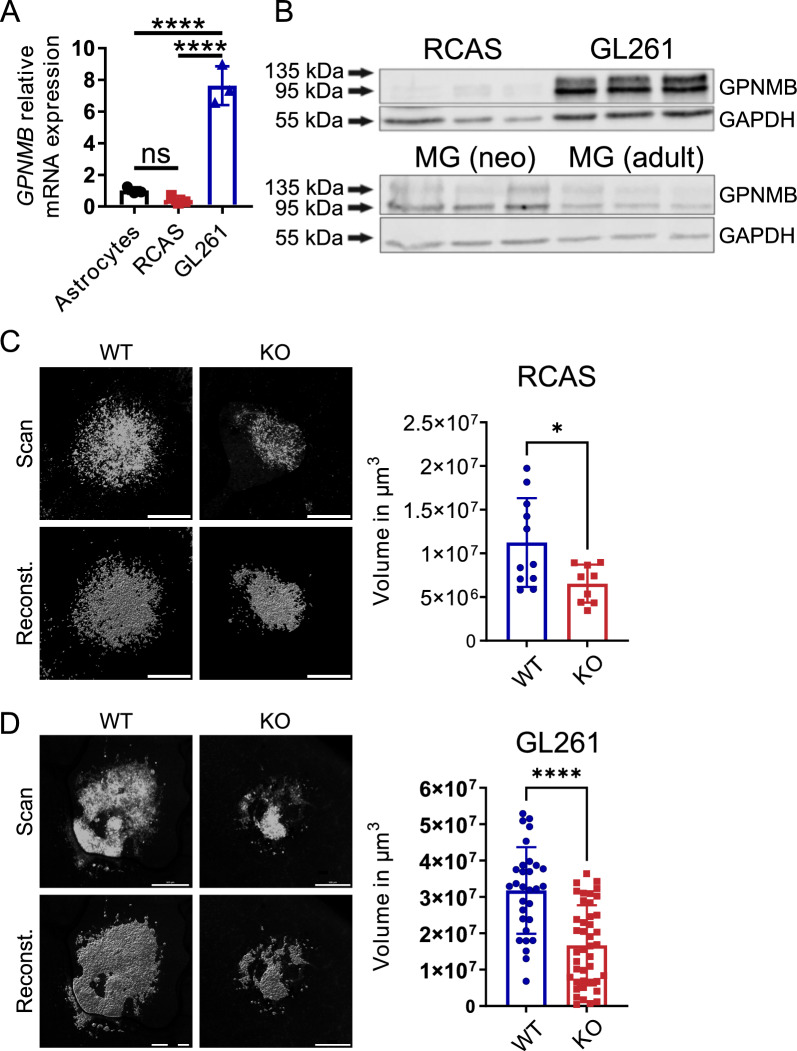
Loss of host-derived GPNMB impairs tumor growth in OBS cultures injected with glioma cells with low and high intrinsic expression of GPNMB. **A** RT-qPCR of GPNMB expression relative to cultured murine astrocytes (n = 3) in cultured RCAS-PDGFb cells (n = 3) and the GL261 glioma cell line (n = 3). Statistical analysis was performed using one-way ANOVA with Tukey’s multiple comparisons test. **B** Western Blot of total GPNMB protein isolated from cultured RCAS-PDGFb, GL261 glioma cells, cultured MG (neonatal) and MG (adult). GAPDH expression serves as a reference. Each column represents an individual batch of culture (n = 3). **C** OBS from WT and KO animals injected with cultured PDGFb-driven RCAS-RFP glioma cells. Images are shown on the left. Top row: Orthogonal view on RFP immunofluorescence based on *z*-stack and tile scanning. Bottom row: representative 3D-reconstruction in Imaris based on *z*-stack and tile scanning. The graph on the right summarizes the volumes (in mm^3^) of WT (n = 11) and KO (n = 9) tumors as determined from the scans. **D** OBS injected with cultured mCherry-GL261 glioma cells. Images are shown on the left. Top row: Orthogonal view on mCherry immunofluorescence based on *z*-stack and tile scanning. Bottom row: representative 3D-reconstruction in Imaris based on *z*-stack and tile scanning. The graph on the right summarizes the volumes (in mm^3^) of WT (n = 30) and KO (n = 42) tumors as determined from the scans. The scale bars represent 500 µm. Statistical analysis was performed using unpaired t-test. Error bars represent SD. ****p < 0.0001, ns, not significant

We used organotypic brain slices (OBS) inoculated with glioma cells to study the impact of GPNMB in microglia without the contribution of infiltrating peripheral cells. Moreover, we employed an additional mouse glioma line, the GL261 line in comparison to the RCAS-PDGFb cells used for in vivo studies. First, we quantified mRNA and protein levels of GPNMB in the RCAS-PDGFb and GL261 murine glioma models and compared the GPNMB levels to those of naïve, primarily cultured astrocytes and microglia of respective neonatal and adult mice as controls. mRNA expression levels of GPNMB were 7.6-times higher in GL261 cells (n = 3) compared to astrocytes (n = 3, *p* ≤ 0.0001). In contrast, cultured PDGFb-driven RCAS glioma cells (≤ 7 passages) showed lower levels of GPNMB expression (n = 3, p = 0.3555) similar to astrocytes. To validate our findings, we used total protein isolates. Little to no expression was found in cultured RCAS-PDGFb cells (n = 3) while GL261 (n = 3) displayed a much higher level of expression. Interestingly, naïve cultured microglia showed low GPNMB expression (Fig. 5A-B). To rule out discrepancies between the in vitro and in vivo expression of GPNMB in the murine glioma cell lines used in this study, we additionally stained tumor brains from GL261 in vivo experiments. In the RCAS- PDGFb (Fig. 2C) and the GL261 (n = 3; Additional file 3; Fig. 3D) murine glioma model, we observed comparable expressions patterns to our in vitro protein isolations. Thus, we could compare glioma cell lines with high and low levels of intrinsic GPNMB expression in our ex vivo models.

To assess the impact of microglial GPNMB in our murine cell lines, we injected RFP expressing RCAS-PDGFb cells or mCherry-GL261 cells into OBS of WT and KO mice. Slices injected with RCAS-PDGFb cells were maintained for 6 days, slices injected with mCherry-GL261 for 4 days. Subsequently, we fixated the slices and labeled the nuclei with HOECHST dye. The slices were then scanned in a confocal microscope and the volume of the RFP fluorescence of RCAS-PDGFb cells (WT: n = 11; KO: n = 9) and the mCherry fluorescence of GL261 cells (WT: n = 30; KO: n = 42) was determined in serial z-stacks. From these data, the volume of the tumor was reconstructed. RCAS-PDGFb glioma cells displayed about 58% less mean volume (*p* = 0.0189) in KO (mean = 0.654 × 10^7^ µm^3^), as compared to WT slices (mean = 1.1 × 10^7^ µm^3^; Fig. 5C). Similarly, GL261 tumors were also 52% (*p* ≤ 0.0001) smaller in KO (mean = 1.67 × 10^7^ µm^3^), than in WT (mean = 3.18 × 10^7^ µm^3^) slices (Fig. 5D). These data suggest that GPMNB derived specifically from microglia and less from the glioma itself promotes glioma growth.

### TAMs are the predominant source of GPNMB in resection tissue from GBM patients

**Fig. 6:**
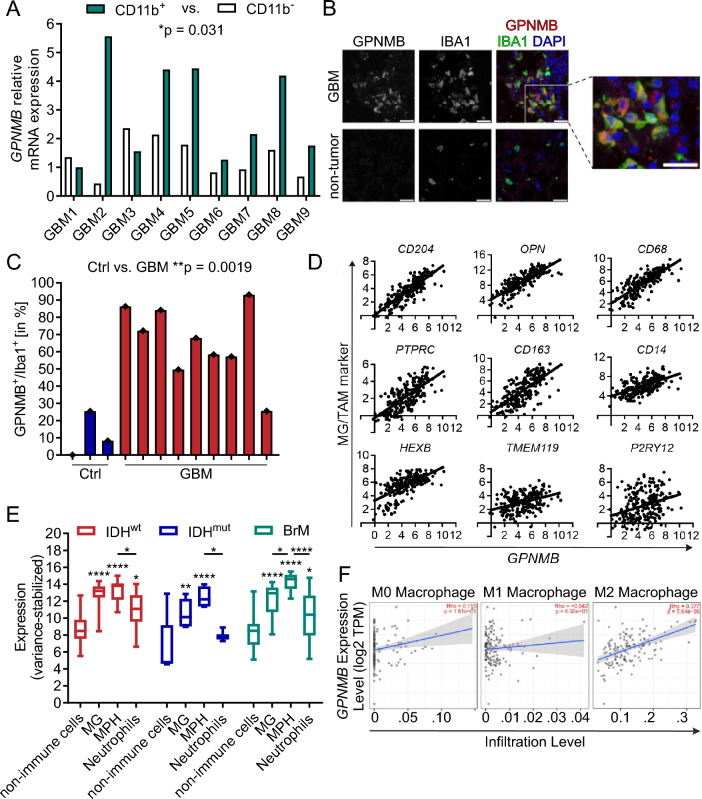
TAMs are the predominant source of GPNMB in resection tissue from GBM patients. **A** RT-qPCR of GPNMB expression in CD11b^+^ and CD11b^−^ cells separated from 9 patients with GBMs. Statistical analysis was performed using paired t-test. **B** Representative staining of patient-derived GBM and non-tumor slices stained for GPNMB (left), IBA1 (middle) and merge with DAPI (right; GPNMB = red, IBA1 = green, DAPI, blue). The image to the right shows a magnified view in the tumor slice. Scale bars, including the magnification, represent 20 µm. **C** Summary of the percentage of IBA1^+^/GPNMB^+^ cells of the samples from GBM (n = 9) and non-tumor (n = 3). Statistical analysis was performed using unpaired t-test. Error bars represent SD. **D** Pearson correlation of tumor-associated macrophage/microglia markers (y-axis) with GPNMB (x-axis). Top: CD204 (r = 0.84; *p* ≤ 0.0001), OPN (r = 0.81; *p* ≤ 0.0001) and CD68 (r = 0.79; *p* ≤ 0.0001). Middle: PTPRC/CD45 (r = 0.70; *p* ≤ 0.0001), CD163 (r = 0.70; *p* ≤ 0.0001) and CD204 (r = 0.59; *p* ≤ 0.0001). Bottom: HEXB (r = 0.64; *p* ≤ 0.0001), TMEM119 (r = 0.37; *p* ≤ 0.0001) and P2RY12 (r = 0.31; *p* ≤ 0.0001). Data derived from all primary GBM samples of the CGGA data set (n = 225). **E** GPNMB gene expression of non-immune cell population (CD45^−^), microglia (MG), macrophages (MPH) and neutrophils in human glioma with IDH wildtype (IDH^wt^), IDH mutant (IDH^mut^) and brain metastasis (BrM) using the Brain Tumor Immune Micro Environment dataset [18]. Unlabeled statistical analysis were performed in comparison to the non-immune cell population with 2way ANOVA and Tukey's multiple comparisons test. Error bars represent min to max. *p < 0.05, **p < 0.01, ***p < 0.001, ****p < 0.0001. **F** The Rho value of correlation between uncommitted (M0, left), pro-inflammatory (M1, middle) and anti-inflammatory (M2, right) macrophage infiltration level (based on TIMER algorithm) and GPNMB gene expression

To determine the expression of human GPNMB in CD11b^+^ and CD11b^−^ cells, we analyzed 9 IDH^wt^ human GBM samples obtained from surgical resection. The tissue was dissociated, and the cells were MACS-sorted into a CD11b^+^ fraction and a CD11b^−^ fraction. We performed RT-qPCR for GPNMB RNA expression in these cell fractions. Our findings show that GPNMB RNA expression is, in average, 3-times higher inCD11b^+^ cells compared to CD11b^−^ cells (Fig. 6A).

To further analyze the expression of GPNMB in glioma tissue, we stained slices from GBM and trauma/non-tumor patients with antibodies against IBA1 and GPNMB. We detected diffuse staining of GPNMB within the tissue with increased expression levels associated to IBA1^+^ cells. GPNMB^+^/IBA1^−^ cells were also detected, but less abundant. Compared to GBM tissue, there was less and diffuse or even no detectable, expression of GPNMB in non-tumor samples (Fig. 6B). Counting of GPNMB^+^/IBA1^+^ cells in each tissue specimen resulted in a mean percentage of 11.26% in non-tumor controls (Ctrl) and 66.06% in GBM slices (*p* = 0.0019; Fig. 6C). Furthermore, we found a significant correlation between the total GPNMB^+^ and IBA1^+^ cells in non-tumor (r = 0.72; *p* = 0.0288) and GBM tissue (r = 0.85; *p* ≤ 0.0001; Additional file 4; Fig. 4A).

We used the published bulk sequencing data of The Cancer Genome Atlas (TCGA) and Chinese Glioma Genome Atlas (CGGA) databases to analyze the expression level of GPNMB in human glioma tissue and distinguished between primary IDH wildtype (IDH^wt^) and IDH mutant (IDH^mut^) tumors and brain metastasis (BrM). The TCGA data set showed significantly higher expression of GPNMB in GBM compared to non-tumor tissue. Both, the TCGA and the CGGA data set showed significantly higher expression of GPNMB in IDH^wt^ compared to IDH^mut^ GBM. BrM display similar expression levels to IDHwt GBM and significantly higher expression with respect to IDH^mut^ GBM (Additional file 4; Fig. 4B-C).

Using the Chinese Glioma Genome Atlas (CGGA) data set, we correlated GPNMB expression with the TAM marker CD204 and OPN, the macrophage/monocytic marker CD68 and CD14, the leukocytic marker PTRC/CD45, the immunosuppressive macrophage marker CD163 and microglia specific marker Hexosaminidase Subunit Beta (HEXB), Transmembrane Protein 119 (TMEM119) and purinergic receptor P2Y12 (P2RY12). All these markers were positively correlated with the expression of GPNMB (Fig. 6D). In contrast, the expression of glial fibrillary acidic protein (GFAP) as an astrocyte marker, oligodendrocyte transcription factor (OLIG2) as an oligodendrocyte lineage marker and EGFR, Cyclin Dependent Kinase Inhibitor 2A (CDKN2A), Cyclin-dependent kinase 4 (CDK4) and Glioma-Associated Oncogene Family Zinc Finger 1 (GLI1) as glioma marker did not show a significant correlation with the expression of GPNMB (Additional file 4; Fig. 4D).

To further validate these findings, we used the FACs-presorted bulk gene expression data set published by Klemm et al. [18] GPNMB expression was analyzed in non-immune cells (CD45^−^), cells designated as microglia and macrophages, and neutrophils pre-sorted innate immune cell populations, stratified into respective glioma entities, namely IDH^wt^, IDH^mut^ and BrM. All entities showed the predominant GPNMB expression in microglia and macrophages similar as found in our murine experiments. We also found significant levels of GPNMB in neutrophils of IDH^wt^ and BrM (Fig. 6E).

Utilizing the CIBERSORT-ABS [40] (Score of arbitrary units that reflects the absolute proportion of each cell type) method based on TCGA GBM RNA-seq dataset on the TIMER platform [37], we found a significant correlation between GPNMB and anti-inflammatory, macrophage infiltration (right; Rho = 0.38; *p* ≤ 0.0001), but none compared to uncommitted (middle; Rho = 0.12; *p* = 0.181) and pro-inflammatory (left; Rho = − 0.04; *p* = 0.630) macrophages (Fig. 6F).

### High GPNMB expression in GBM are negatively prognostic for the disease course and positively correlated for the expression of immune checkpoint markers

**Fig. 7:**
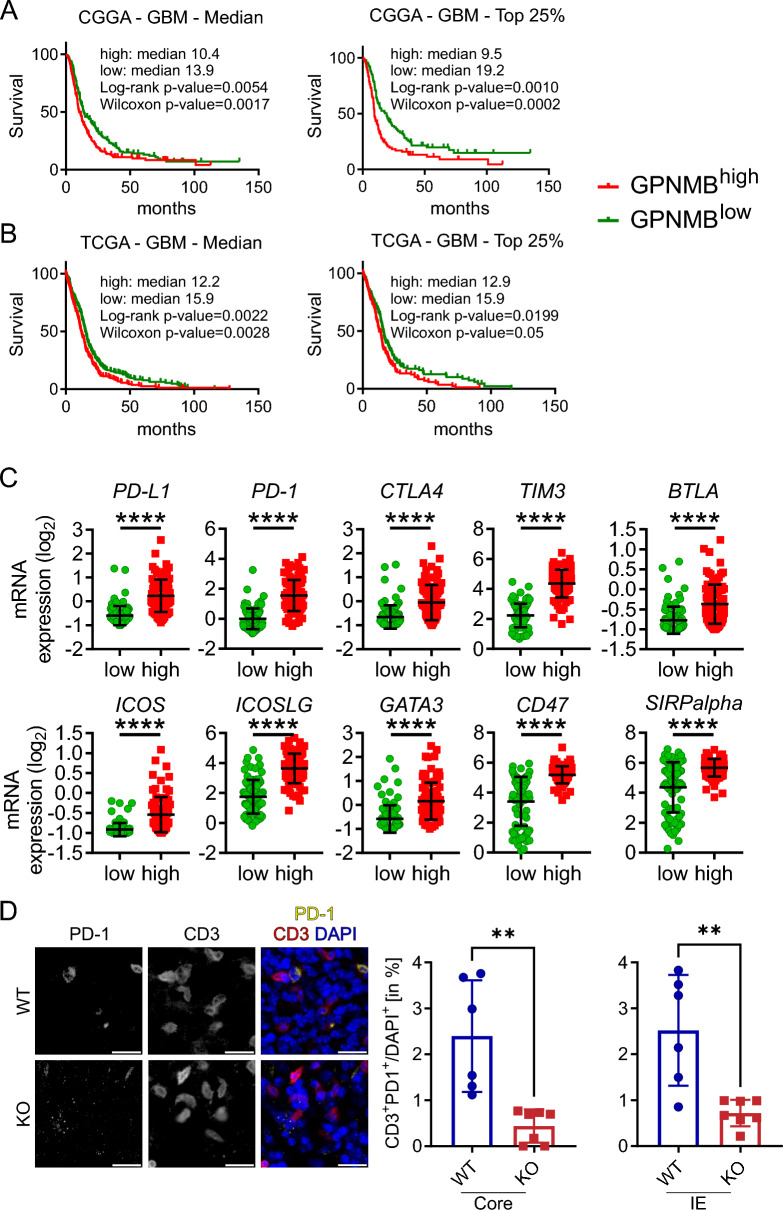
High GPNMB expression in GBM are negatively prognostic for the disease course and positively correlated for the expression of immune checkpoint markers. **A** Kaplan–Meier survival curves of GBM patients based on GPNMB expression in the CGGA data set. Left: Cutoff set by median GPNMB expression into GPNMB^high^ (n = 188, events = 162) and GPNMB^low^ (n = 189, events = 152). Right: Cutoff by top/low 25% GPNMB expression set into GPNMB^high^ (top 25%; n = 94, events = 81) and GPNMB^low^ (low 25%; n = 94, events = 73). **B** Kaplan–Meier survival estimates of GBM patients based on GPNMB expression in the TCGA data set. Left: Cutoff set by median GPNMB expression into GPNMB^high^ (n = 258, events = 223) and GPNMB^low^ (n = 255, events = 212). Right: Cutoff by top/low 25% GPNMB expression set into GPNMB^high^ (top 25%; n = 128, events = 114) and GPNMB^low^ (low 25%; n = 128, events = 106). Statistical analysis was performed using Log-rank (Mantel–Cox) test, Gehan-Breslow-Wilcoxon test and Hazard Ratio (Mantel–Haenszel). **C** Gene expression of immune checkpoint markers (Top: PD-L1, PD-1, CTLA4, TIM3 and BTLA; Bottom: ICOS, ICOSLG, GATA3, CD47 and SIRP-alpha) in GPNMB^high^ (top 25%; n = 94) and GPNMB^low^ (low 25%; n = 94) primary GBM. Data from CGGA. **D** Representative core region staining of PD-1 (left), CD3 (middle) and merge with DAPI (right; PD-1 = yellow, CD3 = red, DAPI = blue) of brain slices from tumor-bearing WT and KO. Scale bar represents 20 µm. The graphs (left, core: WT n = 6, KO n = 7; right, IE: WT n = 6, KO n = 7) show the summary data from WT and KO mice. Statistical analysis was performed using unpaired t-test. Error bars represent SD. *p < 0.05, **p < 0.01, ***p < 0.001, ****p < 0.0001

To assess the prognostic value in GBM, we analyzed data from the CGGA and the TCGA database, separating survival curves into two groups based on GPNMB high and low expression. The first expression cutoff point set to the median, showed significant negative influence on the survival of primary GBM patients in the CGGA (GPNMB^high^: median survival = 10.4 months; GPNMB^low^: median survival: 13.9; HR = 0.72, 0.58–0.91; Log-rank: *p* value = 0.0054; Wilcoxon: *p* value = 0.0017) and TCGA (GPNMB^high^: median survival = 12.2 months; GPNMB^low^: median survival = 15.9; HR = 0.74, 0.62–0.90; Log-rank: *p* value = 0.0022; Wilcoxon: *p* value = 0.0028) data set. This effect was even more pronounced with a cutoff point set to the top 25% expression levels in high vs. low expressing GPNMB samples in the CGGA (GPNMB^high^: median survival = 9.5 months; GPNMB^low^: median survival = 19.2; HR = 0.58, 0.42–0.8; Log-rank: *p* value = 0.0010; Wilcoxon: *p* value = 0.0002) and TCGA (GPNMB^high^: median survival = 12.9 months; GPNMB^low^: median survival = 15.9; HR = 0.73, 0.55–0.95; Log-rank: *p* value = 0.0199; Wilcoxon: *p* value = 0.05) data set (Fig. 7A-B).

Using a range of established innate and adaptive immune checkpoint markers in glioma, namely Programmed cell death 1 ligand 1 (PD-L1), Programmed cell death protein 1 (PD-1), cytotoxic T-lymphocyte-associated Protein 4 (CTLA4), T-cell immunoglobulin and mucin-domain containing-3 (TIM3), B- and T-lymphocyte attenuator (BTLA), inducible T Cell Costimulator (ICOS), inducible T cell Costimulatory ligand (ICOSLG), GATA-binding Protein 3 (GATA3), CD47 and Signal-regulatory protein alpha (SIRP-α), we compared their expression in top 25% high vs. low GPNMB GBM using the data from the CGGA GBM database. All markers were consistently higher expressed in GPNMB^high^ GBM (Fig. 7C).

To validate these findings in our in vivo model and further address the functional state of T cells in WT against KO, we co-labelled tumor slices for CD3 and PD-1. In the core region and the invasive edge of GPNMB-WT tumors, we detected higher levels of PD-1^+^ T cells (Core: WT = 2.4% ± 1.22% SD vs. KO = 0.44% ± 0.36% SD; *p* = 0.0018; Invasive edge: WT = 2.5% ± 1.2% SD vs. KO = 0.72% ± 0.29% SD; *p* = 0.0027) compared to the tumor tissue of KO (Fig. 7D).

### Tissue expression of CD44 is affected by GPNMB in the RCAS-PDGFb model and associated with GPNMB expression in human GBM

**Fig. 8:**
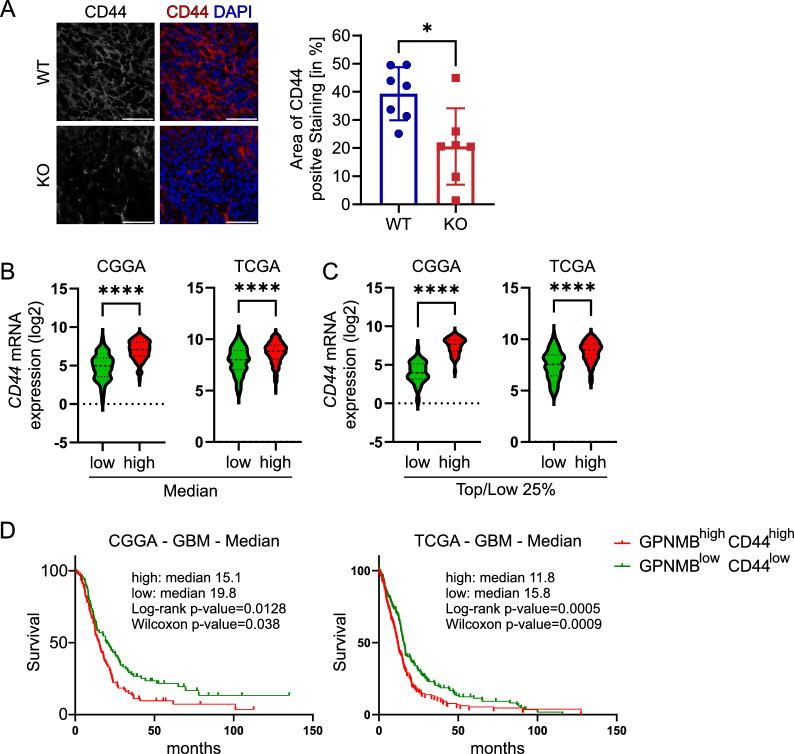
Tissue expression of CD44 is affected by GPNMB in the RCAS-PDGFb model and associated with GPNMB expression in human GBM. **A** Representative staining of CD44 (left) and merge with DAPI (right; CD44 = red, DAPI = blue) of brain slices from tumor-bearing WT and KO. Scale bar represents 50 µm. The graph on the right shows the summary data of the area of CD44 staining (in % of total area) from WT (n = 7) and KO (n = 6) mice. **B** Left: Gene expression of CD44 (Cutoff: median) in GPNMB^high^ (n = 111) and GPNMB^low^ (n = 111) primary GBM. Data from CGGA. Right: Gene expression of CD44 (Cutoff: median) in GPNMB^high^ (n = 262) and GPNMB^low^ (n = 263) primary GBM. Data from TCGA. **C** Left: Gene expression of CD44 in GPNMB^high^ (top 25%; n = 55) and GPNMB^low^ (low 25%; n = 54) primary GBM. Data from CGGA. Right: Gene expression of CD44 in GPNMB^high^ (top 25%; n = 130) and GPNMB^low^ (low 25%; n = 131) primary GBM. Data from TCGA. Statistical analysis was performed using unpaired t-test. Error bars represent SD. **D** Kaplan–Meier survival curves of GBM patients based on GPNMB expression in the CGGA (left) and TCGA (right) data set. Cutoff set by median of CD44 and GPNMB expression into GPNMB^high^CD44^high^ (CGGA: n = 87, events = 77; TCGA: n = 169, events = 149) and GPNMB^low^CD44^low^ (CGGA: n = 86, events = 66; TCGA: n = 170, events = 137). *p < 0.05, **p < 0.01, ***p < 0.001, ****p < 0.0001

CD44 has been previously described as a receptor for GPNMB. We therefore stained CD44 in WT and KO mice. In the WT TME, we observed an overall dense expression pattern, while KO tumors shown less staining intensity. In the RFP^+^ tumor sites, we analyzed the relative area covered by CD44 labelling. We detected significant higher labelling in WT tumors, than in KO tumors (WT = 39.29% ± 9.42% SD vs. KO = 20.57% ± 13.57% SD; *p* = 0.0111; Fig. 8A).

To support this possible interaction with GPNMB and CD44 in human GBM, we separated GPNMB expression in primary GBM by the median into high vs. low expression levels and determined the CD44 expression. GPNMB^high^ GBM show a significant higher CD44 expression than GPNMB^low^ GBM. This effect was even more pronounced when separating tumors by the top 25% and low 25% expression levels of GPNMB (Fig. 8B-C).

Lastly, to explore the importance of GPNMB and CD44 co-expression in primary GBM we analyzed the respective survival curves based on GPNMB and CD44 expression in the CGGA and TCGA data set. The co-expression showed significant negative influence on the survival of primary GBM patients in both data sets (CGGA: GPNMB^high^CD44^high^: median survival = 15.1 months; GPNMB^low^CD44^low^: median survival = 19.8; HR = 0.65, 0.47–0.91; Log-rank: *p* value = 0.0128; Wilcoxon: *p* value = 0.038; TCGA: GPNMB^high^CD44^high^: median survival = 11.8 months; GPNMB^low^CD44^low^: median survival: 15.8; HR = 0.66, 0.52–0.83; Log-rank: *p* value = 0.0005; Wilcoxon: *p* value  = 0.0009) (Fig. 8D).

## Discussion

In the present study, we provide four major findings: (1) Host derived GPNMB is essential for promoting formation and proliferation of gliomas in experimental murine glioma models. (2) GPNMB in murine and human gliomas is predominantly expressed by innate immune cells, especially TAMs. (3) Loss of host-derived GPNMB generates a pro-inflammatory tumor innate and adaptive cellular immune microenvironment. (4) High expression of GPNMB is a negative prognostic factor for patient survival and GPNMB high tumors co-express several immune checkpoint markers. Taken together, these results imply a detrimental role of host-derived GPNMB in modelling the immune cell composition through TAMs in the TME of high-grade glioma.

Several studies have previously linked GPNMB to the promotion of glioma cell motility, cell growth and angiogenesis [30, 31]. Furthermore, GPNMB has been identified as a ligand of CD44, a receptor for hyaluronic acid, which has been shown in GBM to contribute to proliferation, invasion and therapy resistance [22, 24, 41]. Our results emphasize the importance of the glioma environment for the tumor promoting effect of GPNMB. Indeed, GPNMB has been reported to impair T cell proliferation, activation and extravasation by binding Syndecan-4, a mechanism relevant in immunity [25–28, 42–44]. This activity of GPNMB could explain our in vivo observation, namely the reduced levels of Foxp3^+^ T regs and PD-1^+^ T cells in the glioma tissue combined with increased levels of cytotoxic cells, CD8^+^ cells and proliferating T cells.

In normal, non-tumor brain tissue of murine and human origin, the expression level of GPNMB is low. Innate immune cell infiltration, such as TAMs and neutrophils, contribute in TME of gliomas to higher level of GPNMB. These results are in line with the finding that GPNMB is expressed by macrophages in murine and human brain tissue [16, 18, 45]. While the expression of GPNMB in macrophages is well established, GPNMB^+^ expression in neutrophils, as we have found in our study, has not yet been described.

Within the glioma, we also found a population of GPNMB^+^/IBA1^−^ cells. This could be astrocytes or a subgroup of glioma cells. For breast and Head and Neck Squamous Cell carcinoma it has been reported that a subgroup of cancer stem cells express GPNMB and utilize it to persist [22, 46, 47]. Whether GPNMB^+^IBA1^−^ cells are these cancer stem cells remains an open issue.

GPNMB as a membrane protein can be cleaved and the extracellular fragment acts as a paracrine factor to affect neighboring cells for modulating tumorigenesis and immunosuppression. There are, however, also autocrine effects on the GPNMB expressing-cells. It has also been reported that the intracellular domain of GPNMB contributes to macrophage polarization and activity [19]. Suppression of GPNMB expression in the BV2 microglial cell line suppressed the expression of Lipopolysaccharide (LPS) induced Tumor Necrosis Factor alpha (TNF-α), Interleukin-1β (IL-1β), inducible nitric oxide synthase (iNOS), and nitric oxide (NO) levels [48]. Furthermore, knock-down of GPNMB in mouse bone marrow-derived macrophages reduced the secretion of the anti-inflammatory cytokines IL-10 and TGF-β, while the secretion of proinflammatory cytokines IL-1β and TNF-α was increased [49]. Accordingly, overexpression of GPNMB in macrophages promoted the secretion of anti-inflammatory and inhibited the secretion of pro-inflammatory factors [50]. This “innate” regulatory mechanism could explain two observations we made in this study: (1) Increased infiltration of TAMs in the core of KO. (2) Increased antigen-presentation of KO-TAMs. How significant this mechanism is for promotion of tumor growth and impairment of T-cells needs to be addressed in further experiments.

Another still understudied effect of the KO model is the increased density and soma volume of microglia in the naïve and age-matched KO mice. Our data could indicate a more “active” innate surveillance in the KO model. This might explain the restricted localization of glioma cells at the injection site of the KO brains, as the KO immune cells set a “harsher” environment for infiltration and gliomagenesis. These results open the option for an antibody treatment against GPNMB as a potential therapeutic strategy.

### Limitations

The study has several limitations including the usage of a conventional knockout, providing only evidence of tumor growth impairment in murine models as well as not addressing interactions that upregulate GPNMB specifically in respective immune cells.

### Supplementary Information

**Additional file 1; Fig. 1**.**A** Nuclear staining with DAPI of a representative brain slice from a tumor-bearing WT and KO mice. Scale bars represent 2 mm. **B** RFP^+^ fluorescence (grey scaled for visibility) labelling of glioma cells of 3 separate tumor-bearing KO mice. Scale bars represent 500 µm. **C** RFP^+^ fluorescence (grey scaled for visibility) labelling of glioma cells of 3 separate tumor-bearing WT mice. Scale bars represent 500 µm.

**Additional file 2; Fig. 2**. Representative FACS analysis strategy of naïve brain (top), naïve spleen (middle = and tumor-bearing brain tissue (bottom). Single cell solutions were at first sorted for singlets and cell size. For the non-immune cells, we first selected the CD45^−^CD11b^−^ and for lymphocytes the CD45^+^CD11b^−^ population. For microglia/TAMs were defined as CD45^+^CD11b^+^Ly6G^−^Ly6c^−^ population. Monocytes were defined as the CD45^+^CD11b^+^Ly6G^−^Ly6c^+^ population, but were further stratified into Ly6c^low^ and Ly6c^high^. Neutrophils were defined as the CD45^+^CD11b^+^Ly6G^+^Ly6c^+^ population. **B** Cell subtype distribution of the CD45^+^CD11b^+^ population in brain tissue.

**Additional file 3; Fig. 3**. **A** Summary of IBA1^+^ cells in each frame of naïve brain slices of WT (n = 3) and KO (n = 3) mice. Statistical analysis was performed using unpaired t-test. (B) Summary of DAPI^+^ nuclei in each frame of naïve brain slices of WT (n = 3) and KO (n = 3) mice. Statistical analysis was performed using unpaired t-test.** C** IBA1^+^ cell density (normalized to DAPI in %) in brain slices of naïve WT´s (n = 3), tumor WT’s ipsilateral (n = 7) hemisphere (outside of the tumor) and tumor WT´s contralateral (n = 7) hemisphere (outside of the tumor). Statistical analysis was performed using paired t-test between tumor ipsilateral and contralateral hemisphere (*p* = 0.0433). Error bars represent SD. *p < 0.05, **p < 0.01, ns, not significant. **D** Brain slices from GL261 tumor-bearing GPNMB WT mice stained for GPNMB (left), IBA1 (middle) and merge with DAPI (right; GPNMB =red, IBA1 = green, DAPI = blue). Scale bar represents 50 µm.

**Additional file 4; Fig. 4**. **A** Pearson correlation of total GPNMB^+^ cells (y-axis) and total IBA1^+^ cells (x-axis) in one frame. Each dot represents 1 frame. Three distinct areas were pooled per sample of non-tumor (n = 3) and GBM (n = 9). **B** Gene expression of GPNMB comparing human GBM separated into IDH^wt^, IDH^mut^ and brain metastasis (BrM) against non-tumor brain tissue obtained from the TCGA GBM HU-133A database. **C** Gene expression of GPNMB comparing human GBM separated into IDH^wt^, IDH^mut^ and BrM tissue obtained from the CGGA database. **D** Pearson correlation of astrocyte (Glial fibrillary acidic protein, GFAP), oligodendrocyte lineage (Oligodendrocyte transcription factor, OLIG2) and tumor (Epidermal growth factor receptor, EGFR; Cyclin Dependent Kinase Inhibitor 2A, CDKN2A; Cyclin-dependent kinase 4, CDK4; Glioma-Associated Oncogene Family Zinc Finger 1, GLI1) markers (y-axis) with GPNMB (x-axis). Top: GFAP (r = 0.03; *p* = 0.6846), EGFR (r = 0.11; *p* = 0.0114), OLIG2 (r = − 0.25; *p* = 0.0002). Bottom: CDKN2A (r = 0.04; *p* = 0.5424), CDK4 (r = 0.20; *p* = 0.0022), GLI1 (r = 0.15; *p* = 0.0248). Data derived from all primary GBM samples of the CGGA data set (n = 225). ***p < 0.001, ****p < 0.0001.

## Data Availability

The data supporting the findings of this study are available from the corresponding author upon request.

## Abbreviations

ADAM10: A disintegrin and metalloproteinase domain-containing protein 10
BrM: Brain metastasis
BTLA: B- and T-lymphocyte attenuator
CCL18: CC motif chemokine ligand 18
CDK4: Cyclin-dependent kinase 4
CDKN2A: Cyclin dependent kinase inhibitor 2A
CGGA: Chinese Glioma Genome Atlas
CTLA4: Cytotoxic T-lymphocyte-associated Protein 4
DAPI: 6-Diamidino-2-phenylindole
ECM: Extracellular matrix
EGFR: Epidermal growth factor receptor
FCS: Fetal calf serum
GATA3: GATA-bindende protein 3
GBM: Glioblastoma multiforme
GFAP: Glial fibrillary acidic protein
GLI1: Glioma-associated oncogene family zinc finger 1
GPNMB: Glycoprotein nonmetastatic melanoma protein B
Gzmb: Granzyme B
HEXB: Hexosaminidase subunit beta
IE: Invasive edge
ICOS: Inducible T cell costimulator
ICOSLG: Inducible T cell costimulatory ligand
IDH^mut^: Isocitrate dehydrogenase mutant
IDH^wt^: Isocitrate dehydrogenase wildtype
IL-1β: Interleukin-1β
IL-10: Interleukin-10
iNOS: Inducible nitric oxide synthase
KD: Knock-down
LPS: Lipopolysaccharide
MACS: Magnetic-activated cell sorting
MHCII: Major histocompatibility complex II
MMP14: Matrix metalloproteinase-14
MMP2: Matrix metalloproteinase-2
MMP9: Matrix metalloproteinase-9
NO: Nitric oxide
OBS: Organotypic brain slice
OLIG2: Oligodendrocyte transcription factor
OPN: Osteopontin
PBS: Phosphate buffered salt solution
PD-1: Programmed cell death protein 1
PD-L1: Programmed cell death 1 ligand 1
PFA: Paraformaldehyde
P2RY12: Purinergic receptor P2Y12
SD: Standard deviation
SIRP- α: Signal-regulatory protein alpha
TAMs: Tumor-associated microglia and blood-derived macrophages
TBP: TATA-box binding-protein
TCGA: The Cancer Genome Atlas
TGF-ß: Transforming growth factor β
TIM3: T-cell immunoglobulin and mucin-domain containing-3
TME: Tumor microenvironment
TMEM119: Transmembrane protein 119
TNC: Tenascin-C
TNF-α: Tumor necrosis factor alpha
VEGFR: Vascular endothelial growth factor receptor
WT: Wildtype
