# Supplementary figures and images for: Tumor associated microglia/macrophages utilize GPNMB to promote tumor growth and alter immune cell infiltration in glioma
Source: Acta Neuropathol Commun. 2024 Apr 2;12:50. doi: 10.1186/s40478-024-01754-7 (PMC10985997; doi:10.1186/s40478-024-01754-7)

A

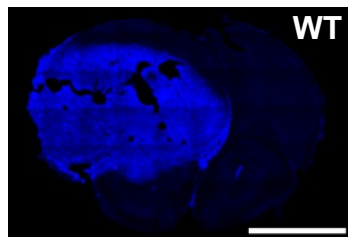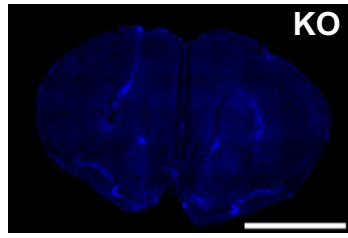

B

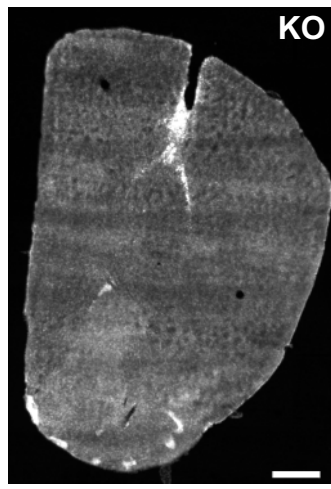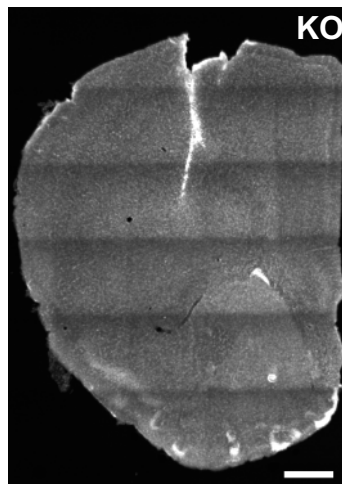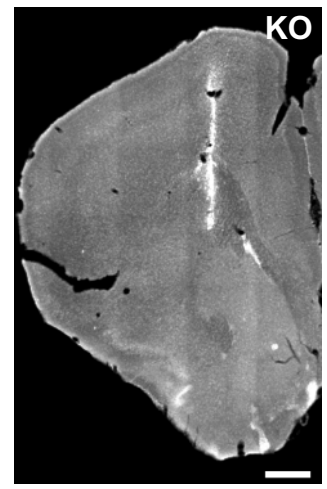

C

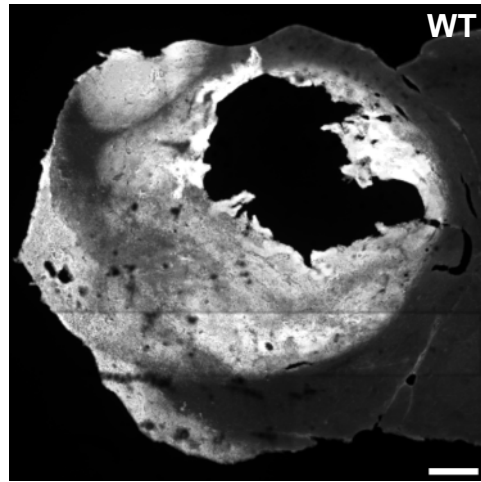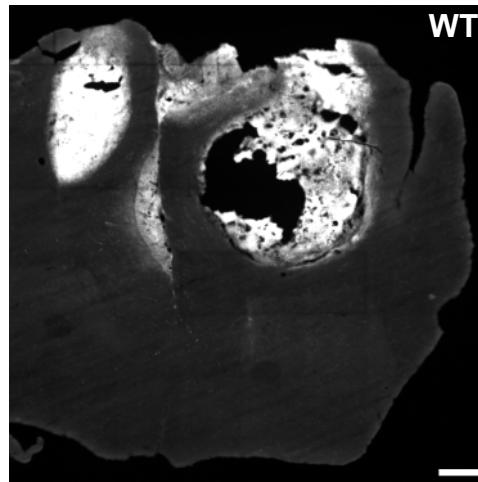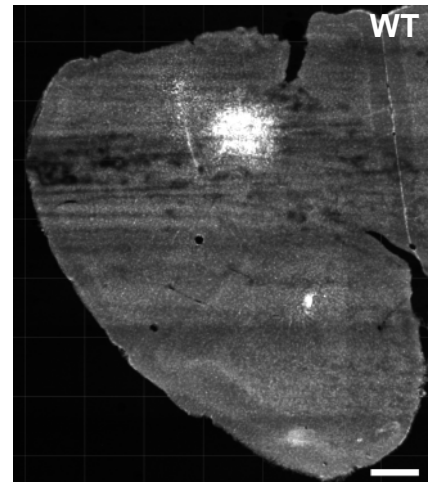

Supplement: Supplementary file 1 — Additional file 1; Fig. 1.A Nuclear staining with DAPI of a representative brain slice from a tumor-bearing WT and KO mice. Scale bars represent 2 mm. B RFP+ fluorescence (grey scaled for visibility) labelling of glioma cells of 3 separate tumor-bearing KO mice. Scale bars represent 500 µm. C RFP+ fluorescence (grey scaled for visibility) labelling of glioma cells of 3 separate tumor-bearing WT mice. Scale bars represent 500 µm. [file 40478_2024_1754_MOESM1_ESM.pdf]

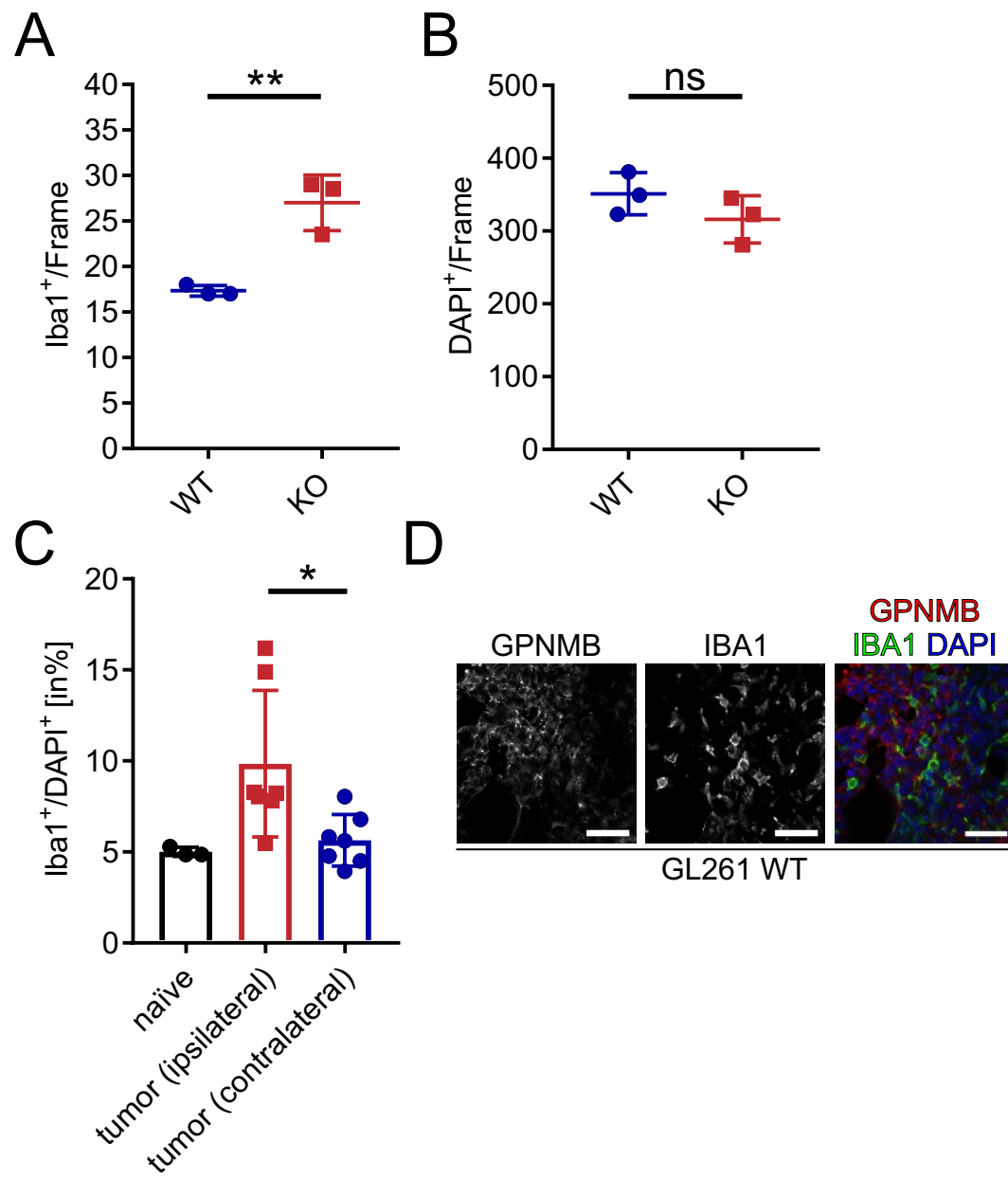

Supplement: Supplementary file 3 — Additional file 3; Fig. 3. A Summary of IBA1+ cells in each frame of naïve brain slices of WT (n = 3) and KO (n = 3) mice. Statistical analysis was performed using unpaired t-test. (B) Summary of DAPI+ nuclei in each frame of naïve brain slices of WT (n = 3) and KO (n = 3) mice. Statistical analysis was performed using unpaired t-test. C IBA1+ cell density (normalized to DAPI in %) in brain slices of naïve WT´s (n = 3), tumor WT’s ipsilateral (n = 7) hemisphere (outside of the tumor) and tumor WT´s contralateral (n = 7) hemisphere (outside of the tumor). Statistical analysis was performed using paired t-test between tumor ipsilateral and contralateral hemisphere (p = 0.0433). Error bars represent SD. *p < 0.05, **p < 0.01, ns, not significant. D Brain slices from GL261 tumor-bearing GPNMB WT mice stained for GPNMB (left), IBA1 (middle) and merge with DAPI (right; GPNMB =red, IBA1 = green, DAPI = blue). Scale bar represents 50 µm. [file 40478_2024_1754_MOESM3_ESM.pdf]

A

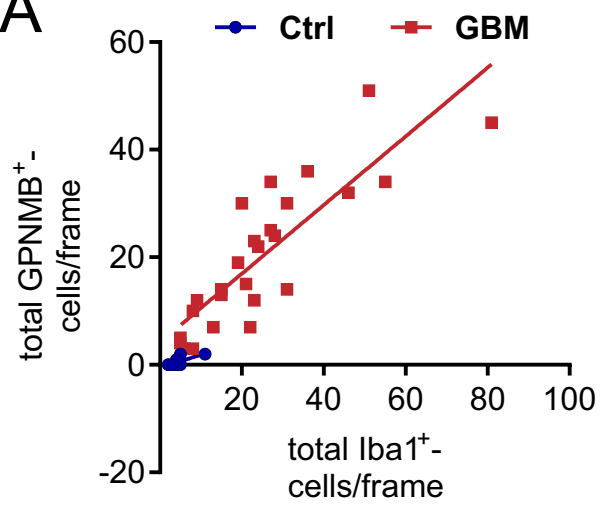

B

CGGA - GBM

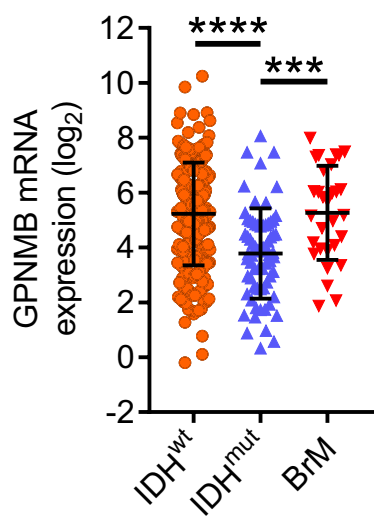

C

TCGA - GBM

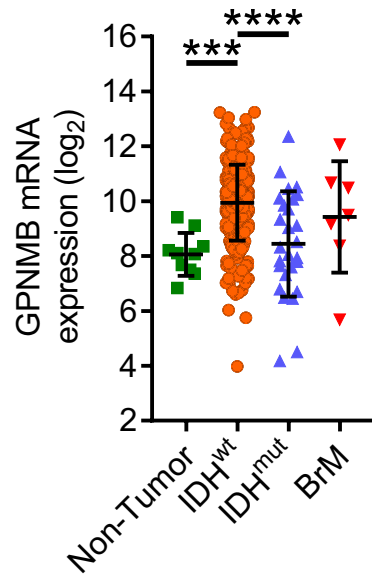

D

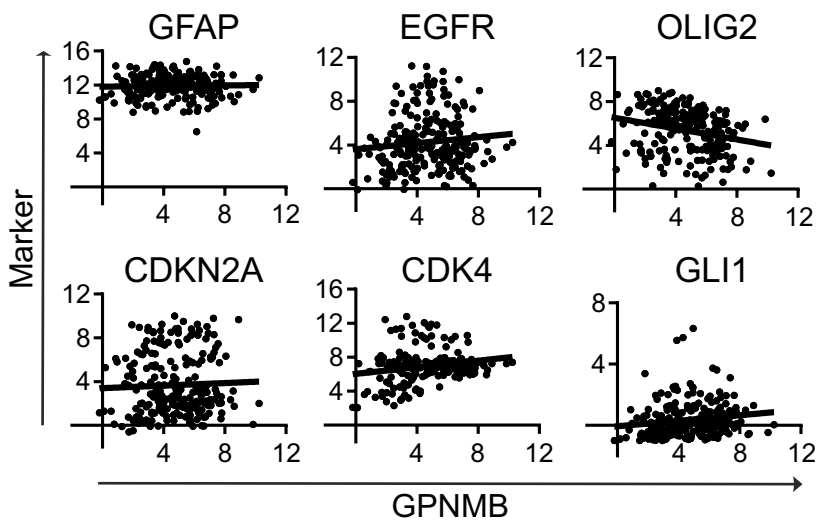

Supplement: Supplementary file 4 — Additional file 4; Fig. 4. A Pearson correlation of total GPNMB+ cells (y-axis) and total IBA1+ cells (x-axis) in one frame. Each dot represents 1 frame. Three distinct areas were pooled per sample of non-tumor (n = 3) and GBM (n = 9). B Gene expression of GPNMB comparing human GBM separated into IDHwt, IDHmut and brain metastasis (BrM) against non-tumor brain tissue obtained from the TCGA GBM HU-133A database. C Gene expression of GPNMB comparing human GBM separated into IDHwt, IDHmut and BrM tissue obtained from the CGGA database. D Pearson correlation of astrocyte (Glial fibrillary acidic protein, GFAP), oligodendrocyte lineage (Oligodendrocyte transcription factor, OLIG2) and tumor (Epidermal growth factor receptor, EGFR; Cyclin Dependent Kinase Inhibitor 2A, CDKN2A; Cyclin-dependent kinase 4, CDK4; Glioma-Associated Oncogene Family Zinc Finger 1, GLI1) markers (y-axis) with GPNMB (x-axis). Top: GFAP (r = 0.03; p = 0.6846), EGFR (r = 0.11; p = 0.0114), OLIG2 (r = − 0.25; p = 0.0002). Bottom: CDKN2A (r = 0.04; p = 0.5424), CDK4 (r = 0.20; p = 0.0022), GLI1 (r = 0.15; p = 0.0248). Data derived from all primary GBM samples of the CGGA data set (n = 225). ***p < 0.001, ****p < 0.0001. [file 40478_2024_1754_MOESM4_ESM.pdf]
