# Supplementary material for: Tumor associated microglia/macrophages utilize GPNMB to promote tumor growth and alter immune cell infiltration in glioma
Source: Acta Neuropathol Commun. 2024 Apr 2;12:50. doi: 10.1186/s40478-024-01754-7 (PMC10985997; doi:10.1186/s40478-024-01754-7)

Supplementary Figure 2

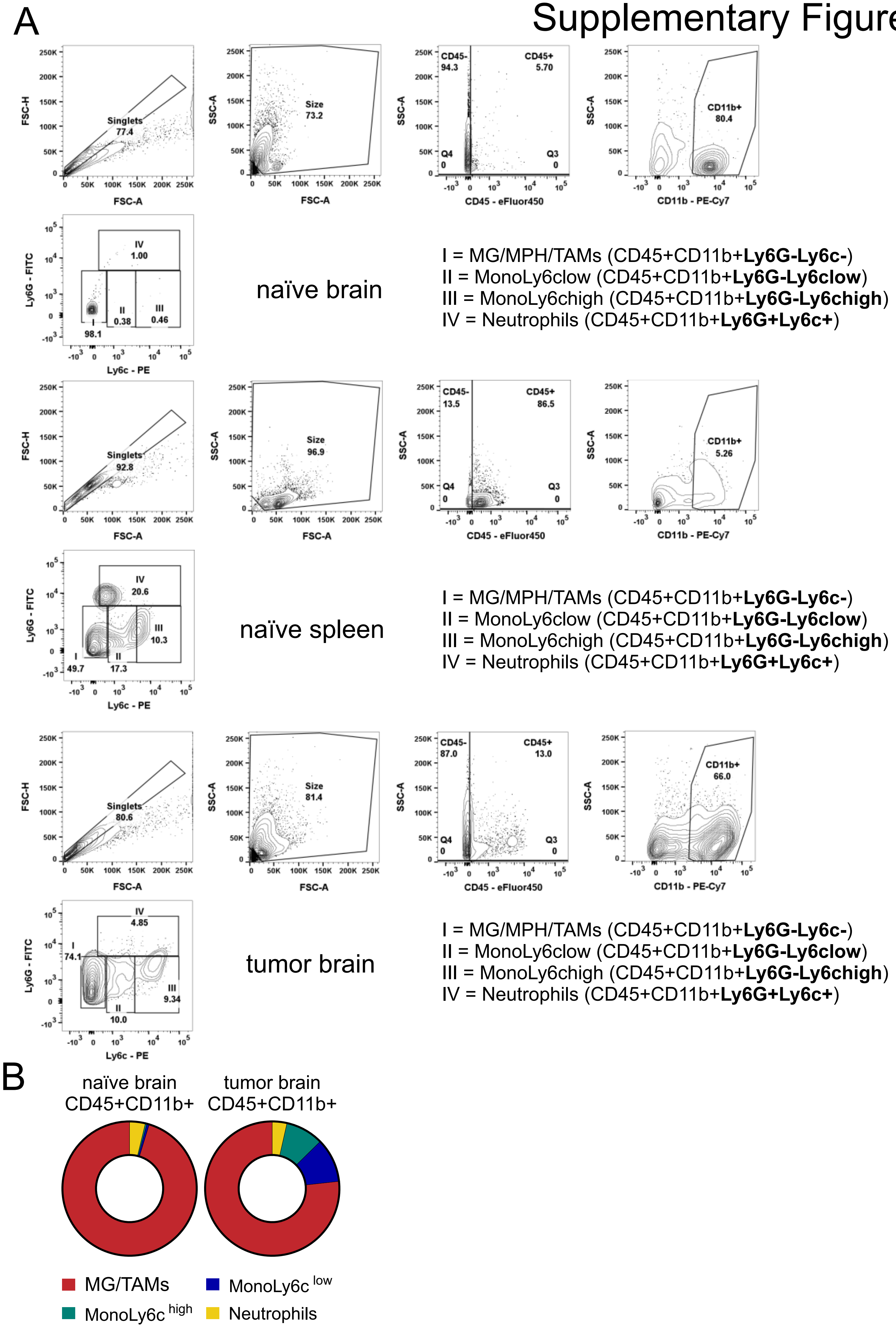

Supplement: Supplementary file 2 — Additional file 2; Fig. 2. Representative FACS analysis strategy of naïve brain (top), naïve spleen (middle = and tumor-bearing brain tissue (bottom). Single cell solutions were at first sorted for singlets and cell size. For the non-immune cells, we first selected the CD45−CD11b− and for lymphocytes the CD45+CD11b− population. For microglia/TAMs were defined as CD45+CD11b+Ly6G−Ly6c− population. Monocytes were defined as the CD45+CD11b+Ly6G−Ly6c+ population, but were further stratified into Ly6clow and Ly6chigh. Neutrophils were defined as the CD45+CD11b+Ly6G+Ly6c+ population. B Cell subtype distribution of the CD45+CD11b+ population in brain tissue. [file 40478_2024_1754_MOESM2_ESM.pdf]
